# A host-specific diaminobutyrate aminotransferase contributes to symbiotic performance, homoserine metabolism, and competitiveness in the *Rhizobium leguminosarum*/*Pisum sativum* system

**DOI:** 10.3389/fmicb.2023.1182563

**Published:** 2023-05-16

**Authors:** Marta Ballesteros-Gutiérrez, Marta Albareda, Coral Barbas, Ángeles López-Gonzálvez, Michael F. Dunn, José M. Palacios

**Affiliations:** ^1^Centro de Biotecnología y Genómica de Plantas (CBGP, UPM-INIA/CSIC), Consejo Superior de Investigaciones Científicas, Instituto Nacional de Investigación y Tecnología Agraria y Alimentaria, Universidad Politécnica de Madrid, Pozuelo de Alarcón, Spain; ^2^Departamento de Biotecnología-Biología Vegetal, Escuela Técnica Superior de Ingeniería Agronómica, Alimentaria y de Biosistemas, Universidad Politécnica de Madrid, Madrid, Spain; ^3^Facultad de Farmacia, Center for Metabolomics and Bioanalysis (CEMBIO), Universidad San Pablo-CEU, Boadilla del Monte, Spain; ^4^Programa de Genómica Funcional de Procariotes, Centro de Ciencias Genómicas-Universidad Nacional Autónoma de México, Cuernavaca, Mexico

**Keywords:** nitrogen fixation, pantothenate auxotrophy, symbiosis, bacteroid, nodulation

## Abstract

*Rhizobium leguminosarum* bv. viciae (*Rlv*) UPM791 effectively nodulates pea and lentil, but bacteroids contain a number of proteins differentially expressed depending on the host. One of these host-dependent proteins (C189) is similar to a **d**i**a**mino**b**utyr**a**te-2-oxoglutarate **a**mino**t**ransferase (DABA-AT). DABA-AT activity was demonstrated with cell extracts and with purified protein, so C189 was renamed as Dat. The *dat* gene was strongly induced in the central, active area of pea nodules, but not in lentil. Mutants defective in *dat* were impaired in symbiotic performance with pea plants, exhibiting reduced shoot dry weight, smaller nodules, and a lower competitiveness for nodulation. In contrast, there were no significant differences between mutant and wild-type in symbiosis with lentil plants. A comparative metabolomic approach using cell-free extracts from bacteroids induced in pea and lentil showed significant differences among the strains in pea bacteroids whereas no significant differences were found in lentil. Targeted metabolomic analysis revealed that the *dat* mutation abolished the presence of 2,4-diaminobutyrate (DABA) in pea nodules, indicating that DABA-AT reaction is oriented toward the production of DABA from L-aspartate semialdehyde. This analysis also showed the presence of L-homoserine, a likely source of aspartate semialdehyde, in pea bacteroids but not in those induced in lentil. The *dat* mutant showed impaired growth when cells were grown with L-homoserine as nitrogen source. Inclusion of DABA or L-homoserine as N source suppressed pantothenate auxotropy in *Rlv* UPM791, suggesting DABA as source of the pantothenate precursor β-alanine. These data indicate that *Rlv* UPM791 Dat enzyme is part of an adaptation mechanism of this bacterium to a homoserine-rich environment such as pea nodule and rhizosphere.

## Introduction

Symbiotic nitrogen fixation by the legume-rhizobia system is the single most important contribution to biological nitrogen fixation in terrestrial habitats (Zhang et al., 2020), and legumes are the second most important group of crops in economic relevance in agriculture (FAO, 2021). Their high value is derived from the production of protein-rich seeds and forages without the requirement of nitrogen fertilization due to their association with rhizobia. The establishment of functional legume root nodules is the result of a sophisticated interaction between plant and bacteria involving the exchange of chemical signals (plant flavonoids and bacterial lipo-chito-oligosaccharides, LPS and EPS) that ensure specificity of the interaction (Perret et al., 2000). Within an indeterminate nodule, such as those from alfalfa, pea, and lentil, an internal zonation is established including infection, interzone and mature areas (Ferguson et al., 2010). Rhizobia in the mature area of the nodule differentiate into bacteroids and express the battery of genes (*nif/fix* and other genes) required for the synthesis and functioning of nitrogenase complex (Poole et al., 2018). Bacteroid metabolism corresponds essentially to a nongrowing state driven by the catabolism of C4-dicarboxylates in an oxygen-depleted environment (Ledermann et al., 2021). The plant supplies carbon sources mainly as malate and succinate, and receives fixed nitrogen as ammonium and, in some cases, alanine (Smith and Emerich, 1993; Lodwig et al., 2004). The bacteroid metabolism shows a high degree of interdependence with that of the plant, with bidirectional exchanges of nutrients, including amino acids, between both partners (Udvardi and Poole, 2013).

Nodulation is the result of essentially single clonal infection events, and a key trait for the successful establishment of a specific strain is the competitiveness to get into legume roots. A key aspect in bacterial competitiveness is the ability to colonize the rhizosphere by detecting, moving toward, and efficiently utilizing root exudates (Mendoza-Suárez et al., 2020). These are complex mixtures of different compounds including sugars, organic acids, amino acids, and vitamins (Badri and Vivanco, 2009) in proportions dependent on plant genotype, plant condition and surrounding microbiota (Dennis et al., 2010), to which bacteria must adapt. Pea root exudates are known to contain L-homoserine (L-Hse, Rovira, 1956), a non-proteinogenic amino acid long known to support growth of *Rlv* strains (van Egeraat, 1975).

The degree of specificity of plant/bacterial components of the symbiosis is variable depending on both partners. *Rhizobium leguminosarum* bv viciae (*Rlv*) UPM791 is a member of the recently described genospecies E within the *Rhizobium leguminosarum* species complex (Young et al., 2021). *Rlv* can establish effective symbiosis with legumes belonging to genera *Pisum*, *Lathyrus*, *Vicia*, *Lens, and Vavilovia* (Young et al., 2021). Although the signaling cascade and mechanisms of root infection, and the general metabolism are likely similar when in association with different legumes (Limpens and Bisseling, 2003; Gage, 2004; Den Herder and Parniske, 2009), the nature of the host affects specific aspects of the symbiosis. It has been shown that symbiotic expression of *Rlv* UPM791 hydrogenase is high in *Vicia villosa*, *V. monanthos, Lathyrus sativus*, and *Pisum sativum,* whereas its expression is rather low in *Lens culinaris* (Brito et al., 2008). Further proteomic analysis of bacteroids induced by *Rlv* UPM791 in pea and lentil nodules showed a significant number of proteins differing in bacteroids from each host (Durán et al., 2021). These host-dependent differences in bacterial strain protein expression indicate the different environments provided by each host plant, to which rhizobia effectively adapt. One of these proteins (C189), was overexpressed in pea vs. lentil bacteroids. C189 is encoded at locus RLV_1844 in the symbiotic plasmid and shows a high similarity to diaminobutyrate-2-oxoglutarate aminotransferases (DABA-AT, EC 2.6.1.76). DABA-ATs are pyridoxal-5-phosphate (PLP)-dependent enzymes that catalyze the reversible transfer of an amino group from glutamate into L-aspartate-β-semialdehyde (L-Asa), resulting in 2,4-diaminobutyrate (DABA) and 2-oxoglutarate. The enzyme has been purified from different bacteria and biochemically characterized as a tetramer able to catalyze the reaction in both directions *in vitro* (Ikai and Yamamoto, 1994; Ono et al., 1998; Hillier et al., 2020), in a manner mechanistically similar to other transaminases such as gamma-aminobutyrate transaminase (Ono et al., 1999; Vandenende et al., 2004; Richter et al., 2019). DABA-AT enzyme is involved in several biological processes in endosymbiotic bacteria, including metabolism of ectoine, and synthesis of siderophores, polyamines, and pantothenate (Ikai and Yamamoto, 1997; Lynch et al., 2001; Becerra-Rivera et al., 2020; Perchat et al., 2022).

Ectoine is a compatible solute frequently produced in *Bacteria* and *Archaea* as cytoprotectant (Talibart et al., 1994; Ikai and Yamamoto, 1997; Ono et al., 1998). Ectoine synthesis is carried out by the products of the well conserved gene cluster *ectABC:* L-2,4-diaminobutyrate acetyltransferase (EctA), diaminobutyrate 2-oxoglutarate aminotransferase (EctB), and ectoine synthase (EctC) that convert aspartate-semialdehyde into ectoine. Ectoine can be used as carbon or nitrogen sources by means of the ectoine degradation cluster *doeABCD*: DoeA, ectoin hydrolase; DoeB, N-acetyl-L-2,4-diaminobutanoate deacetylase; DoeC aspartate-semialdehyde dehydrogenase, and DoeD, diaminobutyrate-2-oxoglutarate transaminase (Schwibbert et al., 2011). In *Sinorhizobium meliloti*, genes for degradation of ectoine (*doeAB* and *eutBC*) are accompanied by those for ectoine uptake (*ehuABCD*) (Jebbar et al., 2005). Some *Rlv* strains (such as *Rlv* 3841) contain the genes *doeABCD*, *eutBC,* and *ehuABCD*, but these gene clusters are not present in the genome of *Rlv* UPM791 (Sánchez-Cañizares et al., 2018), although *doeD*- and *doeB*-like genes are present separately in the RlvUPM791 symbiotic plasmid (RLV_1868 and RLV_1876).

DABA-AT enzyme is involved also in the biosynthesis of siderophores such as rhizobactin 1,021 in *S. meliloti* (Lynch et al., 2001), and pyoverdine in *Pseudomonas aeruginosa PAO1* (Böckmann et al., 1997). However, the genome of *Rlv* UPM791 lacks the accompanying genes required for these routes for siderophore synthesis (Sánchez-Cañizares et al., 2018), thus making the participation of C189 in this process unlikely.

Polyamines (PAs) are widespread aliphatic hydrocarbons that contain two or more amino groups. PAs are involved in diverse physiological processes in bacteria, including growth, stress resistance, motility, rhizobial symbiosis, pathogenesis and biofilm formation (Becerra-Rivera et al., 2020). DABA-AT participates in the production of some of the polyamines (norspermidine and carboxynorspermidine) described in *S. meliloti* (Becerra-Rivera and Dunn, 2019). In this endosymbiotic bacterium PAs contribute to symbiotically relevant phenotypes such as nodulation and nitrogen fixation (Becerra-Rivera et al., 2020).

A recent report (Perchat et al., 2022) has shown that DABA-AT enzyme participates in a pathway for the synthesis of β-alanine, a precursor of pantothenate, in bacterial mutants lacking aspartate decarboxylase (PanD). Although PanD is the only enzyme known to convert aspartate into β-alanine (Dusch et al., 1999), most rhizobia, as well as many α-*Proteobacteria*, are known to lack this enzyme (López-Sámano et al., 2020). *Rlv* is considered an auxotroph for pantothenate and, consistent with this, pantothenate is included as a growth factor in standard recipes for minimal media designed for *Rlv,* such as Rmin or UMS (O’Gara and Shanmugam, 1976; Wheatley et al., 2017).

In this work the role of *Rlv* UPM791 DABA-AT was analyzed in connection with the symbiotic interaction of this bacterium with pea and lentil plants. A DABA-AT deficient mutant was impaired in symbiotic performance and nodulation competitiveness in pea but not in lentil. The same mutant was also affected in the growth of vegetative cells on L-homoserine, a compound present in pea bacteroids but absent in those from lentil. Furthermore, DABA and homoserine were found as sources of pantothenate for this bacterium. The production of this enzyme is likely an adaptation of the bacterium to a homoserine-rich habitat such as pea rhizosphere and nodules.

## Materials and methods

### Bacterial strains, plasmids, and growth conditions

Strains and plasmids used in this study are listed in Supplementary Table 1. *Rlv* UPM791 belongs to genospecies E in a recent update of the phylogeny of what is now considered as a species complex (Young et al., 2021). *R. leguminosarum* strains were routinely grown at 28°C in yeast mannitol broth (YMB; Vincent, 1970), tryptone-yeast extract (TY; Beringer, 1974) or universal minimal salt medium (UMS; Wheatley et al., 2017) with appropriate carbon and nitrogen sources at 10 mM unless otherwise stated. *E. coli* strains DH5α and GM119 were used for standard cloning procedures. *E. coli* strain S17.1 was used for conjugative plasmid transfer between *E. coli* and *R. leguminosarum* and transconjugants were selected in *Rhizobium* minimal medium (Rmin: O’Gara and Shanmugam, 1976). *E. coli* strains were grown in liquid or solid Luria–Bertani (LB) medium (Sambrook, 2001) at 37°C. Antibiotics were used as follows (μg mL^−1^): ampicillin, 100; gentamicin, 10; kanamycin, 20; spectinomycin, 50; and tetracycline, 5 (for *R. leguminosarum*) or 10 (for *E. coli*). Preinocula for determination of growth curves was prepared as follows: rhizobial strains grown for 4 days on YMB plates were used to inoculate 100 mL flasks containing 20 mL of liquid UMS and incubated for 24 h at 200 rpm. Then, cultures were centrifuged and resuspended in UMS without nitrogen, carbon source nor pantothenate. Preinocula were used to inoculate 200 μL of UMS medium with the desired carbon or nitrogen source and the compounds to be tested, to an initial OD_600_ of 0.001. Strains were incubated in 100-well Honeycomb plates (Growth Curves Ltd., Piscataway, New Jersey, United States) with constant, double orbital shaking in a Bioscreen C Pro device (Growth Curves Ltd., Piscataway, New Jersey, United States) at 28°C with OD_600_ measurement intervals of 30 min for 50 h.

### DNA manipulation techniques

DNA manipulations, including purification, restriction enzyme digestions, ligation, agarose gel electrophoresis, PCR amplification, and transformation into *E. coli* cells were carried out by standard protocols (Sambrook, 2001). Oligonucleotides used as primers are listed in Supplementary Table 2. *R. leguminosarum* genomic DNA was extracted from cultures grown in TY using DNeasy Blood and Tissue Kit columns (Qiagen Ltd.) for PCR amplifications. *E. coli* plasmid DNA used for cloning procedures was obtained from cultures grown in LB using NucleoSpin^®^ Plasmid (Macherey-Nagel, Düren, Germany).

Standard PCR amplifications were carried out with *Taq* DNA Polymerase (Roche) in 25 μL total volume. PCR amplifications of DNA fragments above 1 kb, aimed for cloning and fusion PCR, were carried out with PfuUltra II Fusion HS DNA Polymerase (Agilent Technologies) following the manufacturer’s instructions. Colony PCR was used as a quick screen for gene detection directly from *E. coli* and *R. leguminosarum* colonies in 10 μL total volume with 5 μL of NXT Taq PCR Kit (EURx, Gdańsk, Przyrodników).

### Mutant construction

Derivative UPM791 *c189* mutant strain was generated by homologous recombination using the suicide vector pK18*mobsacB* (Schäfer et al., 1994). For this purpose, a first round of PCR reactions amplified 435- and 313-bp DNA fragments covering the up- and downstream regions of *c189* gene, respectively, using primers DABA_AT_P1/DABA_AT_P2 and DABA_AT_P3/DABA_AT_P4, and *Rlv* UPM791 genomic DNA as template. The DNA fragment containing the spectinomycin resistance cassette (Sp^r^) was PCR-amplified with primers spcR_P5_DABA_AT/ spcR_P6_DABA_AT and plasmid pHP45Ω as DNA template. PCR products were linked by their overlapping sequences by a fusion PCR using primers DABA_AT_P1/DABA_AT_P4 and a mixture of the three purified PCR products as templates following the same methodology as described previously (Kuwayama, 2002). The resulting 2,801-bp DNA fragment was cloned in pCR2.1TOPO plasmid rendering plasmid pTPc189 and checked by colony with PCR M13_F_TOPO /M13_R_TOPO and sequencing. Then, the amplified DNA was cloned into the suicide vector pK18*mobsacB* as a *Bam*HI-*Xba*I fragment and the resulting plasmid pK*c189* was introduced into *Rlv* UPM791 strain by conjugation. Homologous recombination by a double crossover even was selected using the *sacB* system (Schäfer et al., 1994). Insertion was verified by PCR using primers DABA_AT_P1/DABA_AT_P4 (discerning between 2,483 bp in the case of mutant and 1944 in the WT) and sequencing analysis. UPM791 *c189*-deficient derivative selected was designated as UPM1458 strain.

Complemented and wild-type over-expression strains for *c189* gene were obtained using the stable plasmid pBBR1MCS-5 to express the gene under its own promoter. A 1,663-bp DNA fragment was obtained containing the entire *c189* gene using primers DABA_AT_COM_F/DABA_AT_COM_R and *Rlv* UPM791 genomic DNA as template. The amplified DNA fragments were digested with *Xba*I and *Hin*dIII enzymes, cloned into pBlueScript vector and checked by PCR and sequencing using PCR M13_F_TOPO /M13_R_TOPO primers. The DNA insert was then recovered as *Xba*I-*Hin*dIII restriction fragment and ligated into the broad host range vector pBBR1MCS-5. The resulting plasmid, pBBRc189 was checked again by PCR and sequencing using specific primers of the vector (M13_F_TOPO/M13_R_TOPO). Then plasmid was introduced into the *Rlv* UPM791 and UPM1458 strains by conjugation. Presence of the vector in *Rlv* strains was checked by PCR amplification.

### Construction of a taurine-inducible expression system for *c189* gene

A 1,707-bp DNA fragment containing the *c189* gene and its native promoter was PCR-amplified with primers DABA_PLMB51_PL_F/DABA_StrepTag_R, the latter primer encoding also for *Strep*Tag II peptide (WSHPQFEK) for in-frame fusion of the tag sequence to the 3’ end of the gene. The resulting PCR product was cloned into pBlueScript vector as *Bam*HI-*Xba*I fragment, checked by colony PCR and sequencing (PCR M13_F_TOPO/M13_R_TOPO). Then, DNA fragments were cloned into vector pLMB51 (Tett et al., 2012) upstream of the promoterless *gusA* reporter gene using the same restriction enzymes, thus resulting in pLMBc189_ST_ plasmid. This plasmid was introduced into the *Rlv* UPM791 and UPM1458 strains by conjugation and putative positive colonies were checked by PCR using pLMB51_Check_F/pLMB51_Check_R primers and sequencing. This vector contains a taurine-inducible promoter upstream of cloning site. To induce expression of C189, bacteria harboring pLMBc189_ST_ plasmid were grown in UMS with 2 mM taurine.

### Plant assays

Pea (*Pisum sativum* L. cv. Frisson) and lentil (*Lens culinaris* cv. Magda) seeds were surface sterilized and pregerminated on 1% water agar plates as previously described (Sánchez-Cañizares et al., 2011). Seedlings were placed under bacteriologically controlled conditions in Leonard jar-type assemblies filled with sterilized vermiculite and with a nitrogen-free plant nutrient solution, and inoculated with 1 mL of rhizobial cultures collected at stationary phase (Ruiz-Argüeso et al., 1978). Plants were grown in a greenhouse adjusted to 18/25°C (night/day) temperatures and 16 h of light/dark photoperiod.

For symbiotic effectiveness assay, 21 days-post-inoculation (dpi) pea plants and 30 dpi lentil plants were harvested to measure shoot dry weight, nodule number and fresh weight. Nitrogenase activity was determined in nodulated roots using the acetylene reduction assay (Ruiz-Argüeso et al., 1978). Shoot dry weight was determined after drying at 60°C for 48 h.

To estimate competitive ability, rhizobia cultures were counted with a Petroff-Hausser counting chamber, and diluted to 10^5^ cells mL^−1^ in Vincent buffer (Vincent, 1970). One milliliter of inoculant containing the strains to be tested mixed in 1:10, 1:1 and 10:1 proportions were used to inoculate pea and lentil seedlings. Twenty-one dpi pea and 30 dpi lentil plants were harvested, and all nodules were collected and stored at −80°C until analyzed. To differentiate between strains, nodules were surface sterilized as described for seeds and bacteria isolated from each nodule were tested for resistance to spectinomycin in YMB. Predicted nodule occupancy was estimated on the basis of colony forming units that were counted by plating the tested mixtures in media with and without antibiotic.

### RNA extraction and RT-qPCR

For total RNA extraction, nodule samples from 21 dpi pea plants and 30 dpi lentil plants were ground in a cold mortar with 500 μL of TRIzol reagent (Sigma-Aldrich) and transferred into a cold microfuge tube. Following incubation for 5 min at RT, 100 μL of chloroform was added, and after mixing, samples were incubated for 3 min at RT and centrifuged (16,000 × *g*, 15 min); RNA was precipitated overnight at −20°C in the presence of 1 μL of glycogen and 250 μL of isopropanol. Following centrifugation (21,300 × *g*, 10 min at 4°C), the pellet was washed with 1 mL of 75% ethanol, centrifuged (16,000 × *g*, 1 min) and resuspended in 40 μL nuclease free water (EURx, Gdańsk, Przyrodników), incubated at 60°C for 5 min, and transferred to ice. Then, 5 μL of DNase buffer, 2.5 μL of Turbo DNase (Invitrogen, Thermo Fisher), and 2.5 μL of RNase-Out (Fisher Scientific) were added to the sample and incubated for 30 min at 37°C. RNA was purified using Tri-Reagent (Life Technologies), treated with DNase turbo (Life Technologies), and cleaned with a RNeasy Mini kit (Qiagen). RNA concentration was quantified with a Nanodrop spectrophotometer and tested for possible DNA contamination by PCR using the RNA samples as templates and primers specific for *rpoD* (rpoD_qPCR_F/rpoD_qPCR_R). RNA integrity was confirmed by electrophoresis in a 1% agarose gel.

cDNA was synthesized from 500 ng of DNA-free RNA using PrimeScript RT reagent Kit (Takara, Saint-Germain-en-Laye, France), supplemented with RNase out (Invitrogen) following manufacturer’s specifications.

Expression analysis was carried out by real-time reverse transcription polymerase chain reaction (RT-qPCR) using a StepOne plus thermocycler (Applied Biosystems, Foster City, CA, United States), the Power SyBR Green master mix (Applied Biosystems) and the primers c189_qPCR_F/c189_qPCR_R for *c189*, hupL*_*qPCR_F/hupL*_*qPCR_R for *hupL*, and gabT*_*qPCR_F/gabT*_*qPCR_R for *gabT* genes.

### Purification of C189-*Strep*Tag II fusion protein

Protein purification was carried out from 3 L of taurine-supplemented UMS culture of *R. leguminosarum* UPM791(pLMBc189_ST_) expressing C189:StrepTagII (C189_ST_) under the control of a taurine-dependent promoter. Bacterial cultures were grown until stationary phase and centrifuged (10,000 × *g*, 30 min). Then pellets were suspended in 10 mL of buffer W (50 mM HEPES), pH 8, 500 mM NaCl and 0.4 mM tris (2-carboxyethyl) phosphine (TECEP) containing a protease inhibitor mixture (Complete-mini; Roche Diagnostics GmbH) and 100 μM pyridoxal phosphate. Cells were disrupted by three passages through a French pressure cell (SLM Aminco, Silver Spring, MD) at 100 MPa, and soluble fractions were cleared of cell debris and membranes by ultracentrifugation at 135,000 × *g* at 4°C for 1 h. The supernatant (soluble extract) was added to a 1-mL StrepTactin Superflow column (IBA, Göttingen, Germany) operated by gravity flow. The column was washed seven times with 1 mL of buffer W to remove unbound proteins, and the tagged protein was eluted by the addition of 3 mL (6 × 500 μL) of buffer W supplemented with 2.5 mM D-desthiobiotin. Eluted fractions were collected, pooled, concentrated using a centrifugal filter device (Amicon Ultra 4 mL, 3 K), and supplemented again with 100 μM pyridoxal phosphate. Final preparation was adjusted to 10% glycerol before storage at −20°C.

### β-Glucuronidase activity assays

β-glucuronidase (GUS) activity was determined in free-living cells grown in UMS media and in bacteroids of *R. leguminosarum* strains as described (Lodwig et al., 2004). GUS activity was determined by measuring the production of *p-*nitrophenol from p-nitrophenyl-β-D-glucuronide substrate with quantitation based on total protein. Values of activity are calculated as OD_420_ min^−1^ (mg protein)^−1^. Protein content of suspensions of vegetative cells and bacteroids was determined by the bicinchoninic acid method (Smith et al., 1985) after alkaline digestion of cells at 95°C in NaOH for 10 min, using bovine serum albumin as standard.

GUS staining of nodule sections was carried out in nodules from 30-day-old pea and 45 day-old lentil plants as previously described (Boivin et al., 1990). Frozen nodules were sliced into 80-μm sections using a Leica VT1200S vibratome. The sections were incubated in staining buffer (50 mM sodium phosphate buffer pH 7; 0.1% Triton X-100; 3.3% Sarkosyl 30%; containing 0.5% X-GlcA) for 1 h. The reaction was stopped by washing the sections with distilled water. Samples were observed in loupe Leica MZ95 with a CCD color Leica DFC 280 camera (1.4 Mpixels).

For iodide staining, nodule sections were washed once with 12% commercial bleach, then once with distilled water, incubated for 1 min in 10% potassium iodide, and washed again with distilled water (Dylan et al., 1986).

### DABA-AT activity assay

For the preparation of cultured cell extracts, 400-ml bacterial cultures grown in UMS to an OD_600_ of 0.5 were harvested by centrifugation (10,000 × *g*, 30 min, 4°C; Avanti J-HC, Beckman), washed with 30 mL of 20 mM phosphate buffer (pH 7.2) and centrifuged again under the same conditions. Bacteroid suspensions were obtained from 0.3 g of nodules as previously described (Leyva et al., 1987). Vegetative and bacteroid cells were suspended in 4 mL breaking buffer (40 mM HEPES, 1 mM DTT at pH 7.0) and disrupted by three passages using a French pressure cell (SLM Aminco, Silver Spring, MD) at 100 MPa. Cell extracts were centrifuged (21,300 × *g*, 25 min, 4°C) and kept on ice until their use. Protein concentrations in the cell extracts ranged between 0.5 and 3 mg mL^−1^.

2,4-diaminobutyrate 2-oxoglutatarate aminotransferase (DABA-AT) activity was measured by quantifying glutamate production in a coupled glutamate dehydrogenase (GDH) assay (Prell et al., 2002). The first reaction was performed in a final volume of 500 μL with 0.1 mM Tris (pH 8.5), 0.1 mM pyridoxal-5-phosphate, 5 mM 2-oxoglutarate, 150 mM 2,4-diaminobutyrate and 250 μL cell extract. The reaction mixture was incubated at 28°C for 90 min, stopped by boiling the mix for 10 min, and centrifuged (10 min, 16,000 × *g*) to eliminate precipitated proteins.

For glutamate dehydrogenase assay, the previous supernatant (500 μL), was brought to 1.5 mL 40 mM hydrazine, 50 mM glycine (pH 7.6), 2.7 mM NAD^+^. Reaction was started by addition of 3 units of GDH (Sigma, G-2626), and incubated for 90 min at 37°C. Glutamate was quantified by measuring the amount of NADH at 340 nm in a spectrophotometer (Ultrospec 3,300 pro, Amersham Biosciences) and comparing to a standard curve (0–200 nmol glutamate), prepared freshly every day in duplicate. As control, samples without GDH enzyme and without DABA for each extract were included.

DABA-AT enzymatic activity was determined also in purified C189_ST_ protein as follows: the first reaction was carried out in a final volume of 100 μL containing 50 mM HEPES (pH 8.5), 500 mM NaCl, 0.4 mM TCEP, 2 mM 2-oxoglutarate, 10 mM DABA, and 0.2 μg/μL purified C189_ST_. Reaction was incubated for 20 min at 28°C, and then stopped by heating the mix 10 min at 95°C. The GDH coupled reaction was as described for cell extracts, but in a final volume of 300 μL and performed in 96-well plates (Costar, Corning, NY, United States).

### Protein gel electrophoresis and western immunoblot

Proteins (60 μg total protein of crude cell extracts) were resolved by standard sodium dodecyl sulfate-polyacrylamide gel electrophoresis (SDS-PAGE) or native PAGE in 8–16% mini-Protean TGX Precast acrylamide gels (Bio-rad Laboratories, Hercules, CA, United States), and transferred to a Polyvinylidene fluoride 0.45-micron filter (Inmobilon^™^-P, Millipore, Bedford, MA, United States) as previously described (Brito et al., 1994). Proteins were detected immunologically using *Strep*Tactin antibody conjugated to alkaline phosphatase (1:2,500; IBA, Göttingen, Germany), and immunoblots were developed with a chromogenic substrate (bromochloroindolyl phosphate-nitro blue tetrazolium) as recommended by the manufacturer (Bio-Rad Laboratories, Inc. Hercules, CA, United States). Protein sizes were estimated by comparing their rates of migration to those from a reference pattern from commercial BlueStar Prestained Protein Ladder (Nippon Genetics, Tokyo, Japan).

### Polyamine analysis by high-performance thin-layer chromatography (HPTLC)

Dansyl (DNS) derivatives of PAs were detected through HPTLC analysis (Becerra-Rivera et al., 2018) of extracts obtained from 48-h UMS cultures pelleted by centrifugation and resuspended to an OD_600_ of 3.0 in fresh UMS. To determine the free PAs in bacteroids, 300 mg nodules (21 dpi plants for pea and 30 dpi for lentil), were used to isolate bacteroids as above mentioned, and the pellet was lyophilized and resuspended in 0.5 mL of 5% (w/v) trichloroacetic acid following the same protocol as for vegetative cells. Plates were visualized under UV light and images captured with a Syngene (Frederick, MD, United States) InGenius imaging system.

### Metabolomic profile

Three hundred mg of nodules were collected from 21 and 30 dpi pea and lentil plants, respectively, and bacteroids were extracted immediately as previous described (Leyva et al., 1987). In order to minimize quenching metabolism, nodules were picked and placed on ice. Bacteroids were prepared in less than 40 min. Once prepared, bacteroid pellets were frozen in liquid nitrogen and kept at −80°C until proteomic determination.

The pellets corresponding to around 10^7^ cells were resuspended in 300 μL of cold methanol and disrupted with an ultrasonic probe (16 burst of 0.5 s per pulse at 80% intensity). After centrifugation at 15,000 × *g*, 15 min, 100 μL of the supernatant was transferred to a new tube and evaporated to dryness in a SpeedVac (Wang et al., 2020). The metabolite extracts were re-suspended in 0.1 mol L^−1^ formic acid containing 0.2 mmol L^−1^ methionine sulfone (internal standard) by 1 min vortex mixing and then centrifuged (15,000 × *g*, 15 min). All samples were analyzed by CE-ESI-MS-TOF with a method based on a previous one developed by CEMBIO group (Moraes et al., 2011) with slight modifications. The system used was a Capillary Electrophoresis System (7100 Agilent) coupled to a time-of-flight mass spectrometry system (Agilent 6224) equipped with an electrospray source. An ISO Pump (1200 Agilent) was used to supply sheath liquid. In brief, separation was under normal polarity with a background electrolyte containing 1.0 mol L^−1^ of formic acid in 10% methanol (v/v) at 20°C. New capillaries were pre-conditioned by flushing successively by 1.0 mol L^−1^ NaOH, MilliQ^®^ water and background electrolyte, 30 min each. Samples were hydrodynamically injected at 50 mbar for 35 s and stacked by injecting background electrolyte at 100 mbar for 10 s. The separation voltage was 30 kV with 25 mbar of internal pressure and analysis time was 30 min. The optimized MS parameters were: fragmentor 125 V, Skimmer 65 V, octopole 750 V, nebulizer pressure 10 psi, drying gas temperature at 200°C and flow rate 10.0 L·min^−1^. The capillary voltage was 3,500 V. Data were acquired in positive Dual-ESI mode with a full scan from m/z 70–1,000 at a rate of 1.36 scan/s. The resulting CE-MS data files were treated with Targeted Feature Extraction tool with Profinder software (B.06.00, Agilent). Peak areas from the extracted ion chromatograms were integrated and revised. These metabolites were previously identified in the samples by comparison of their exact monoisotopic mass and migration time with database house (Mamani-Huanca et al., 2021).

### Statistical analyses

Unless otherwise specified, comparisons of more than two groups were done by ANOVA for multiple comparisons, *post hoc* corrections as indicated in each figure legend. Error bars represent standard deviations of the assay. Statistical analyses are indicated following the level of significance: (****) *p* < 0.0001, (***) *p* < 0.001, (**) *p* < 0.01, (*) *p* < 0.05, ns no statistical significance (*p* > 0.05) All analyses were performed using GraphPad Prism 8 (GraphPad Software) and Metaboanalyst.^1^

## Results

### C189 protein is a diaminobutyrate aminotransferase

Comparison of C189 protein sequence with databases revealed a significant similarity to biochemically characterized DABA aminotransferases, such as those from *Chromohalobacter salexigens* and *Paenibacillus latus* (Richter et al., 2019; Hillier et al., 2020). Apart from a significant general similarity with these proteins (identities of 54.4 and 49.2%, respectively), residues described as specific for DABA aminotransferases (Hillier et al., 2020) were fully conserved in C189 (Supplementary Figure 1). Homologs of C189 with a highly significant level of conservation (over 75% identity) were also found within several members of *Rhizobiaceae* (Supplementary Figure 2).

**Figure 1:**
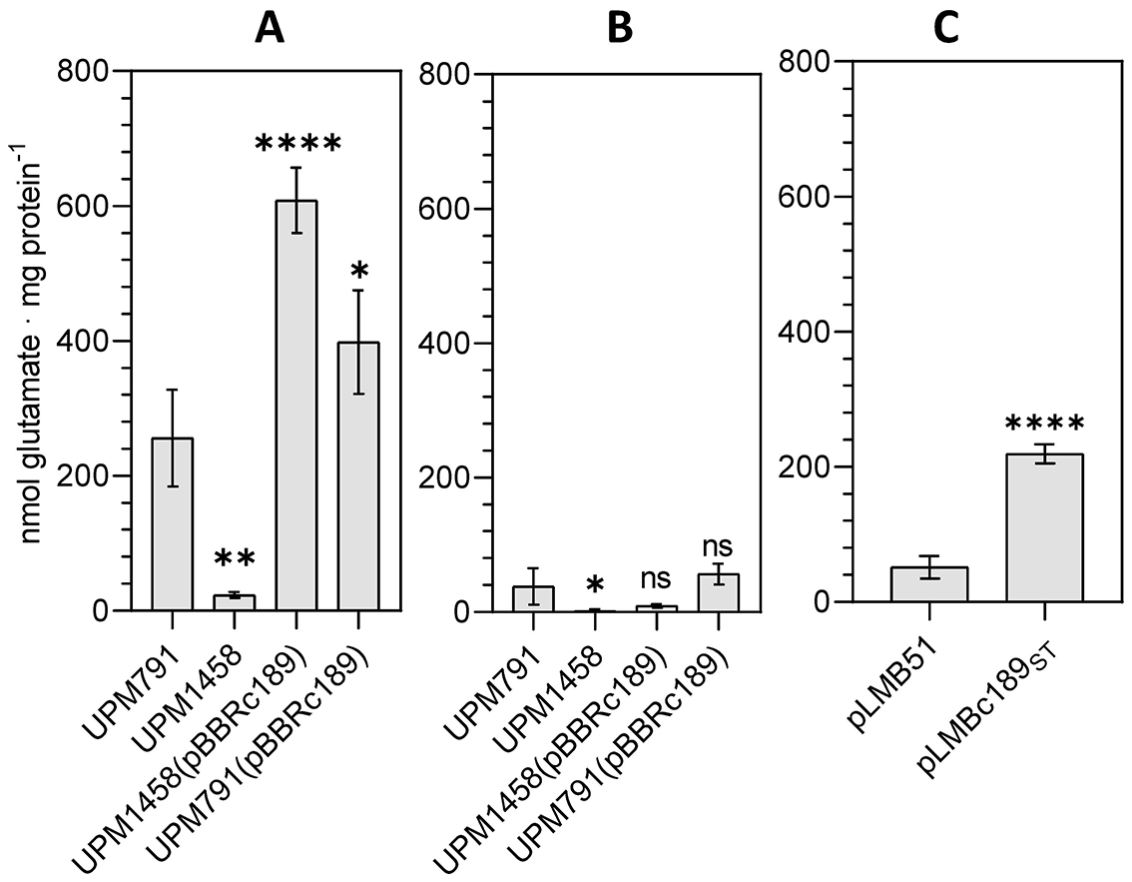
DABA-AT enzymatic activity. **(A,B)** DABA-AT activity of extracts from bacteroids induced by the indicated *Rlv* strains in pea and lentil nodules, respectively. **(C)** Extracts from free-living cells of *Rlv* UPM791 derivatives carrying empty vector pLMB51 or *dat* fusion pLMBc189_ST_, as indicated*,* grown in UMS with 2 mM taurine. Bars correspond to mean value of four replicates. Differences were analyzed by one-way ANOVA with Dunnett’s test for multiple comparisons.****P < 0.0001, **P < 0.01, *P < 0.05; ns, indicates no statistically significant difference with respect the UPM791 values in A and B.

To obtain biochemical evidence of the activity catalyzed by C189 protein, the level of DABA-AT activity in cell-free extracts of pea bacteroids obtained from *Rlv* strain UPM791 was compared to that from its *c189*-deficient derivative UPM1458. In this analysis, significant level of DABA-AT activity was found in *Rlv* UPM791 (Figure 1A). In contrast, the level of activity was greatly reduced in the mutant *Rlv* UPM1458, whereas it was significantly increased in strains incorporating extra copies of *c189* in plasmid pBBRc189. The same analysis revealed very low levels of DABA-AT activity in bacteroids induced by the same strains in nodules from lentil plants (Figure 1B). These data indicate that the presence of C189 protein results in DABA-AT activity in *Rlv* UPM791 pea bacteroids.

DABA-AT activity was also assayed in cell extracts from taurine-induced cultures of the *Rlv* UPM791(pLMBc189_ST_). In these assays, the presence of the *c189* gene resulted in significant increases of DABA-AT activity in the extract as compared to those from cells carrying the empty vector (Figure 1C). These results also indicate that the C-terminal insertion of StrepTag affinity tail in C189_ST_ protein is compatible with the DABA-AT activity of the protein.

**Figure 2:**
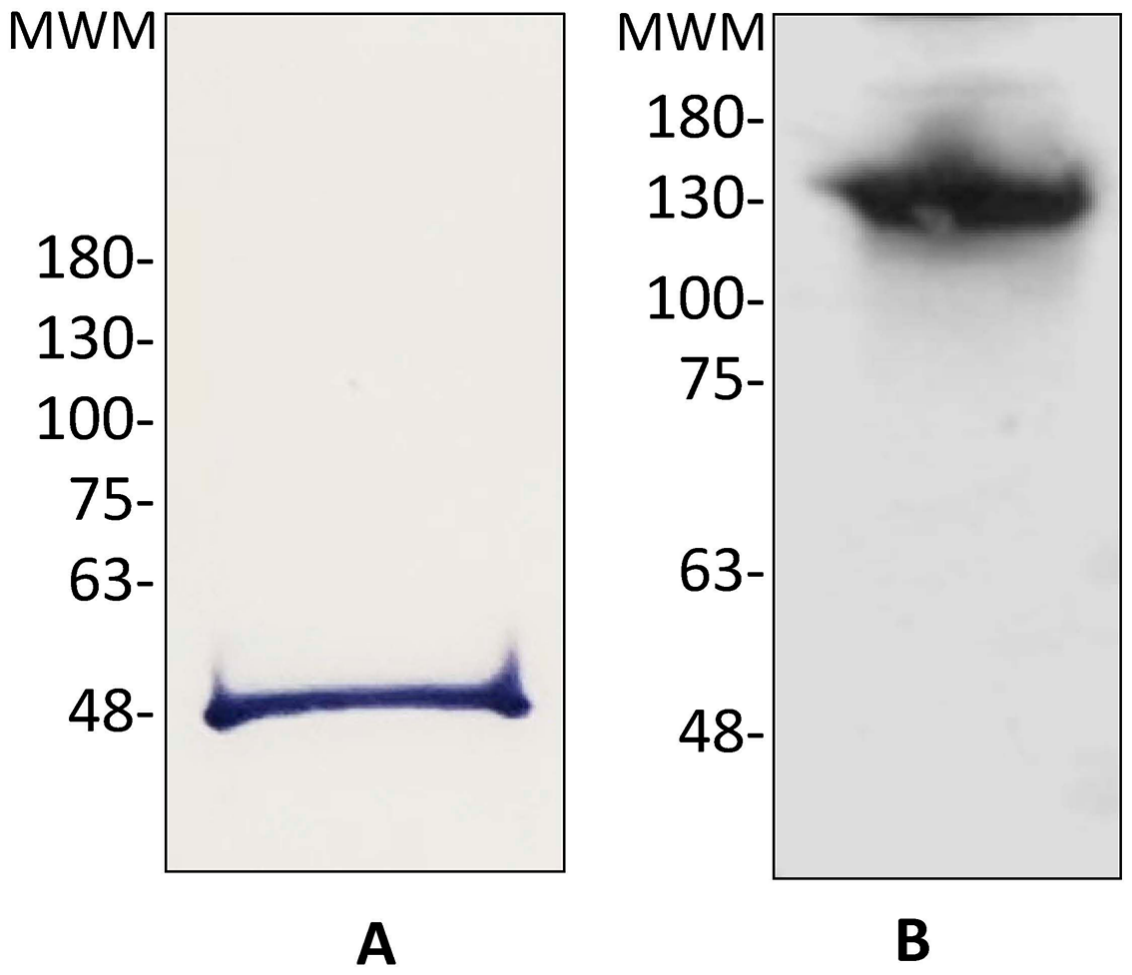
Immunodetection of *Rlv* UPM791 C189_ST_. **(A)** Immunoblot from 12% acrylamide SDS-PAGE containing purified C189_ST_. **(B)** Immunoblot of 8–16% acrylamide native gel containing purified C189_ST._ MWM, location of molecular weight markers, in kDa.

StrepTag tail was utilized to purify the protein from taurine-induced cultures of *Rlv* UPM791(pLMBc189_ST_) (see Materials and methods for details). In these experiments C189_ST_ was concentrated over 28-fold, with a specific DABA-AT activity of *ca*. 5 micromoles of glutamate.h^−1^ (mg prot)^−1^. The purified protein migrated in SDS-PAGE gels as a single band of *ca*. 48 kDa (Figure 2A), in reasonable agreement with the expected size deduced from the sequence of C189_ST_ (47.1 kDa). Analysis of the purified protein in native acrylamide gels revealed that the protein is an oligomer, likely a tetramer (Figure 2B).

As conclusion from all these data, *Rlv* UPM791 C189 is a diaminobutyrate 2-oxoglutarate aminotransferase. Consequently, C189 protein was renamed as Dat, and the corresponding gene *c189* was renamed as *dat*.

### *dat* gene is differentially expressed in the mature zone of pea but not lentil nodules

To ascertain whether the control of the observed host-dependent expression of Dat protein in bacteroids was exerted at the level of transcription, the relative mRNA levels of the *dat* gene were determined by qRT-PCR in pea and lentil nodules. These experiments showed that the level of *dat* transcript in pea was significantly higher than in lentil bacteroids (27.5-fold ±6). In contrast, the transcript levels of the gene encoding an 4-aminobutyrate aminotransferase (GabT, encoded in locus Rlv_7110), a protein that showed comparable levels in both legume hosts in a previous analysis (Durán et al., 2021), were similar in both hosts (0.85-fold ±0.11). Transcripts of the gene encoding the [NiFe] hydrogenase large subunit (*hupL*), a positive control known to be subject to a strong host-dependent regulation (Brito et al., 2008), showed a significant increase in pea vs. lentil nodules (81.8-fold ±11).

**Figure 3:**
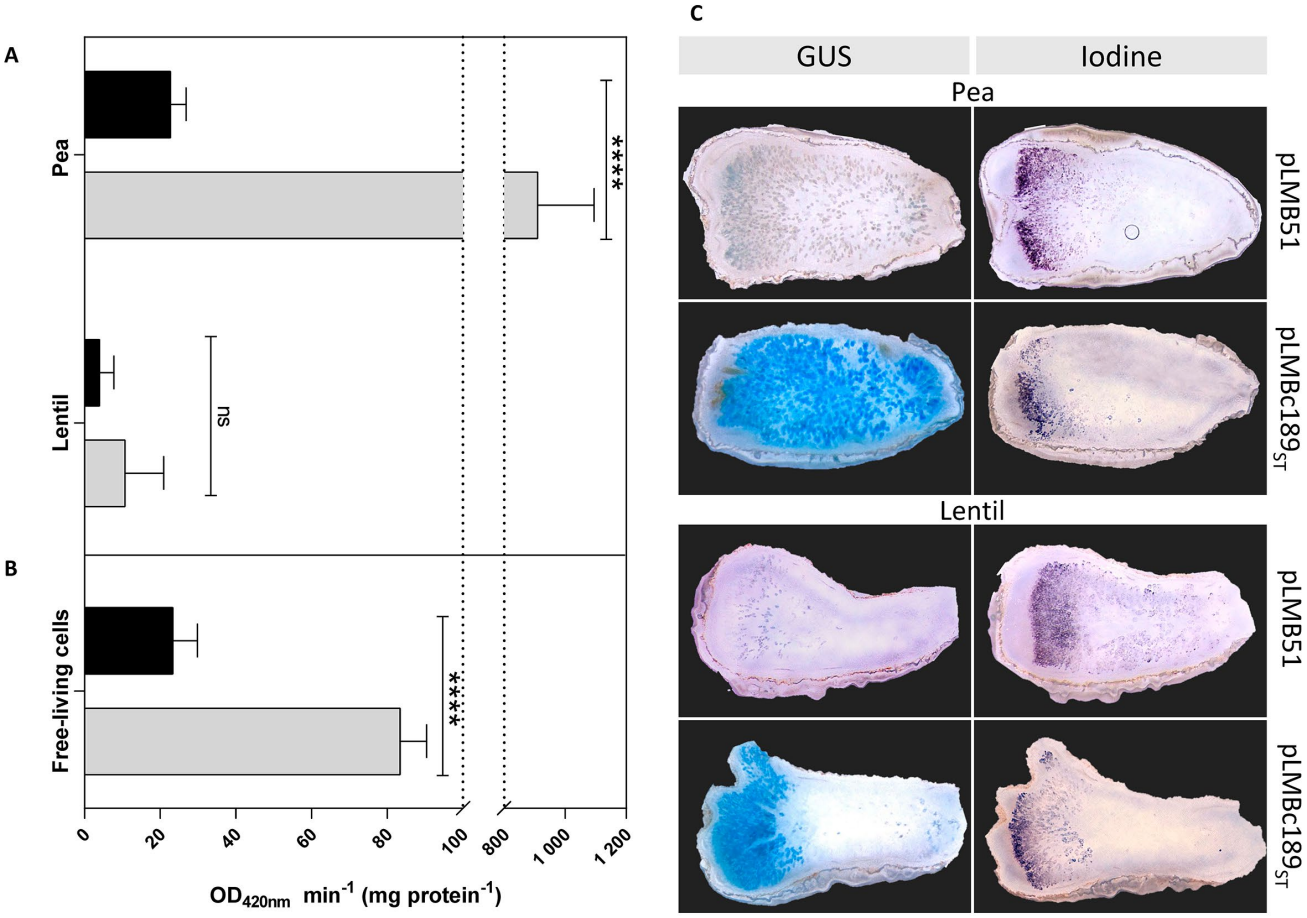
Reporter gene analysis of the expression of *Rlv* UPM791 *dat* gene. β-Glucuronidase (GUS) activities were determined in pea and lentil bacteroids **(A)** and in free-living cells **(B)** carrying empty vector pLMB51 (black bars) or pLMBc189_ST_ (gray bars). GUS activities are the mean of two independent experiments with two replicates each. Differences were analyzed by one-way ANOVA with Bonferroni for multiple comparisons ****P < 0.0001; ns, no statistically significant *P* > 0.05. **(C)** *in-situ* staining of 80-μm longitudinal nodule sections induced by the indicated strains in pea (four top panels) and in lentil (four bottom panels). Panels on the left correspond to GUS staining of nodules produced by *Rlv* UPM791 derivatives carrying empty vector pLMB51 or *dat* fusion pLMBc189_ST_, as indicated. Panels on the right correspond to consecutive sections to those in the left, stained with potassium iodide to reveal starch granules in II-III interzone region.

In order to obtain additional evidence of the host-dependent *dat* expression, the transcriptional fusion of *c189* to β-glucuronidase reporter gene present in plasmid pLMBc189_ST_ was used (see Materials and Methods section). In these experiments, pea and lentil bacteroids of *Rlv* UPM791 derivatives bearing either the empty vector pLMB51 or pLMBc189_ST_ were assayed for reporter activity. As shown in Figure 3A, strains harboring the empty vector exhibited basal levels of reporter activity in bacteroids induced in both legume hosts. In contrast, the level of reporter activity associated with pLMBc189_ST_ in pea bacteroids was high (over 800 units), whereas only basal levels were detected in bacteroids induced by the same strain in lentil. The same constructions were used to ascertain whether *dat* gene was expressed in free-living cells (Figure 3B). In these experiments, low but significant levels (*ca.* 80 units) of reporter activity were observed in cultures of *Rlv* UPM791(pLMBc189_ST_), well over the background level (9 units) observed in cells carrying the empty vector. This level of expression, although significantly lower than that observed in bacteroids, suggests that Dat protein might have a role outside the nodule.

In order to monitor the nodule regions in which *dat* expression takes place, *in-situ* staining for β-glucuronidase activity was carried out in nodule sections. Pea nodules of *Rlv* UPM791(pLBMc189_ST_) showed intense blue staining in the central mature region. In contrast, *Rlv* UPM791 derivatives carrying the empty vector showed no significant signal (Figure 3C). This central region was not stained in lentil nodules, where only peripheral cells surrounding the meristematic region of the nodules were stained in blue. In all sections, the II-III interzone, where bacteroids mature into nitrogen fixing cells, was identified by iodine staining of adjacent nodule sections to detect the characteristic accumulation of starch nodules. The results obtained indicate expression of the *dat* gene in the mature, nitrogen fixing region of pea but not lentil nodules.

### *Rlv dat* contributes to symbiotic performance and competitiveness in pea plants

Since the expression of the *dat* gene was detected in mature bacteroids from pea nodules, the potential effect of the corresponding protein on symbiosis was tested. To do so, the symbiotic performance of wild-type *Rlv* UPM791 was compared with that of *dat*-deleted strain *Rlv* UPM1458. Complemented strains were generated by introduction of a pBBRMCS-based wild-type gene into both the wild-type and mutant strains. These four strains were used as inocula for pea and lentil plants, and their symbiotic performance was determined under controlled conditions using a nutrient solution without nitrogen.

**Figure 4:**
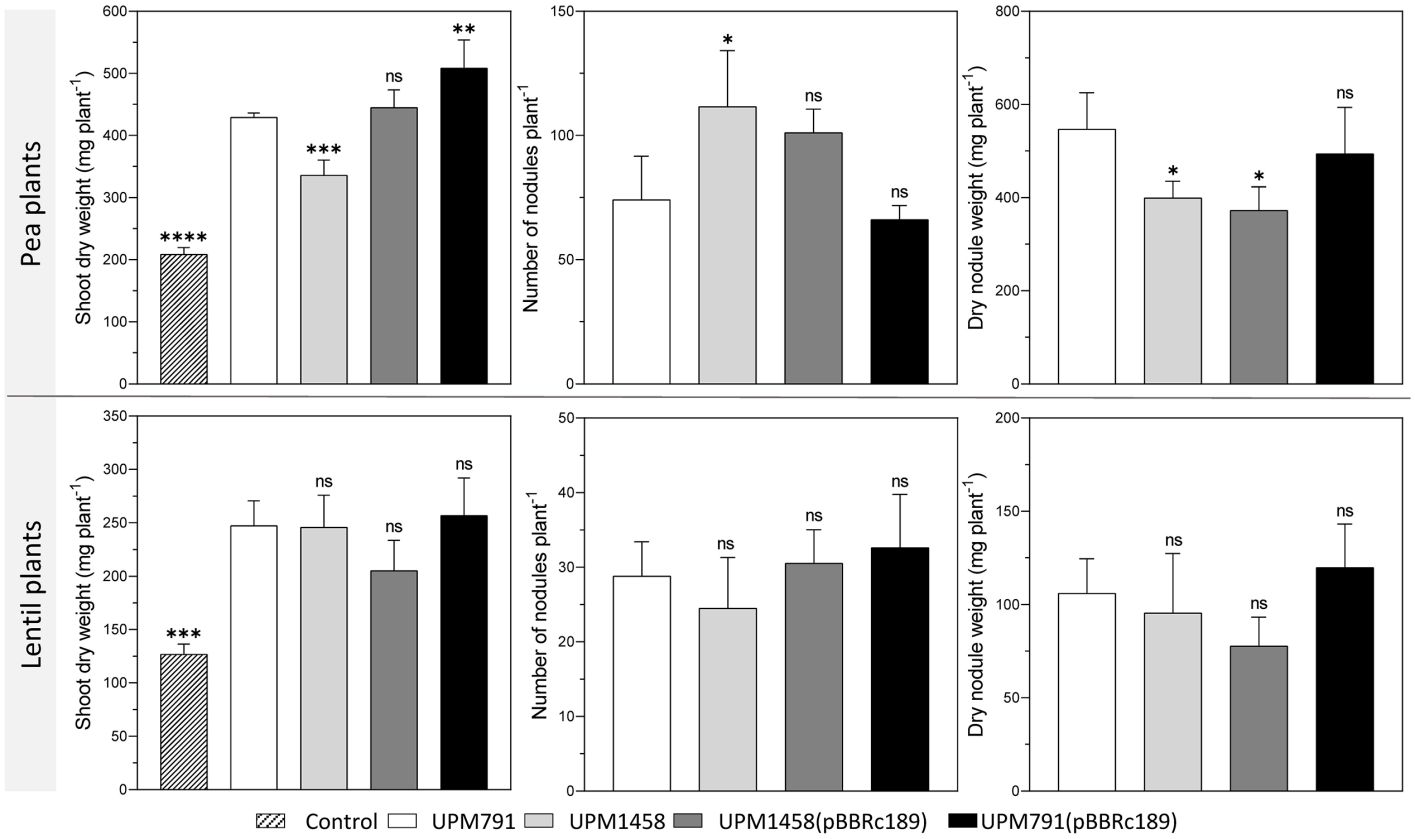
Effect of *dat s*ymbiotic phenotype of *Rlv* in pea and lentil plants. Values of shoot dry weight, nodule number and fresh nodule weight of pea and lentil plants inoculated with *Rlv* UPM791, UPM1458 *(dat* deletion derivative), UPM1458(pBBRc189) (complemented mutant), UPM791(pBBRc189) (over-expression strain) correspond to the averages of four replicates. As a control, non-inoculated plants were used to verify the absence of cross-contamination. Differences were analyzed by one-way ANOVA with Bonferroni for multiple comparisons with respect UPM791 values.****P< 0.0001, ***P < 0.001, **P <0.01, *P < 0.05; ns, indicates no statistically significant difference.

All rhizobial strains included in the assay induced red, nitrogen-fixing nodules on both hosts, resulting in plants that accumulated significantly more dry matter than the uninoculated controls (Figure 4). The comparison between pea plants inoculated by wild-type and *dat*-deficient strains revealed that the mutation was associated with a significant reduction in the biomass accumulated in the shoot (21.7% decrease in dry matter). In this host, the mutant induced smaller nodules, with a significant decrease in nodule fresh weight, along with an increase in nodule number (Figure 4). The introduction of a plasmid bearing a wild-type copy of the gene in the mutant resulted in the reversion of the symbiotic phenotype as regarding shoot dry weight and nodule number. In contrast, no significant differences were observed in the case of lentil plants inoculated with the same strains (Figure 4). These results indicate that the presence of Dat protein contributes to optimal levels of symbiotic performance in pea but not in lentil nodules. In order to ascertain whether the different symbiotic performance was due directly to the nitrogen fixation activity, the level of nitrogenase activity was determined in nodules induced in pea by wild-type and mutant strains. In this assay, nodules from both strains exhibited similar levels of specific acetylene reduction activity [13.4 ± 0.77 and 14.97 ± 2.09 nmoles ethylene· h^−1^ · (g nodule fresh weight)^−1^ in wild-type and mutant strains, respectively].

**Figure 5:**
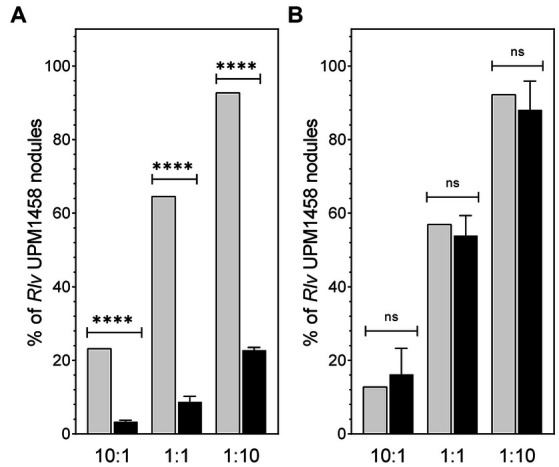
Effect of *dat* on nodulation competitiveness of *Rlv* UPM791 in pea and lentil plants. **(A)** Competitiveness in pea. **(B)** Competitiveness in lentil. Grey bars represent the predicted percentage of nodules calculated from the viable cells count in the mixtures of strains used as inocula. Black bars represent the percentage of spectinomycin-resistant strains (UPM1458) recovered from nodules resulting from the indicated strain combinations. Values are the average of 4 independent replicates, in which all nodules of the plant were analyzed. Standard deviation in viable cells counting was below 10%. Differences were analyzed by χ^2^.****P < 0.0001, ns, indicates no statistically significant difference.

The competitiveness for nodulation is a key trait defining performance of rhizobial strains used as legume inoculants, so the effect of *dat* deletion on the competitive ability to induce nodules in pea and lentil was tested. To this end plants of both legume species were inoculated using 1:1, 10:1, and 1:10 mixtures of UPM791:UPM1458 *Rlv* strains. Scoring of nodules induced by each strain revealed significant reductions (*ca*. 80%) on the number of nodules induced by the mutant in pea as compared with the expected values derived from viable counting of the inocula in the three mixtures (Figure 5A). These data indicate an impairment of the competitiveness associated to the elimination of the *dat* gene on this host. The same competitiveness assay was carried out with mixed inocula in lentil plants. In this case, no significant reduction in the number of nodules induced by the mutant strain was observed, suggesting that wild-type and mutant strains had similar competitiveness in lentil (Figure 5B).

### Analysis of the metabolic role of *dat* gene in *Rhizobium leguminosarum*

The positive role of Dat protein on symbiosis with pea led us to wonder how DABA-AT activity might benefit the symbiotic process. In other bacteria, this enzymatic activity is involved in several cellular processes, including ectoine metabolism, and synthesis of siderophores, PAs and pantothenate. As stated above, the *Rlv* UPM791 genome contains no gene clusters similar to those described for ectoine and siderophore metabolism, so first the potential role of Dat in PA synthesis was tested.

**Figure 6:**
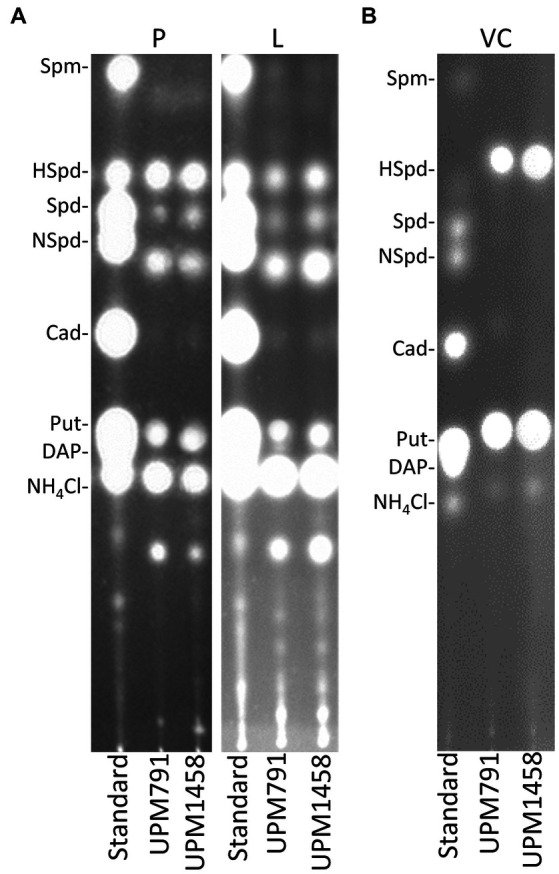
Polyamine content of *Rlv* strains. The pictures show fluorograms of HPTLC-resolved dansyl-derivatized soluble intracellular polyamines present in **(A)** bacteroids from pea (P) and lentil (L) plants, and in **(B)** vegetative cells (VC) of *Rlv* strains UPM791 (wild-type) and UPM1458 (*dat* mutant). In all cases pure polyamine standards were loaded in the left lane.

PAs are known to play a significant role in the *Rhizobium*-legume symbiosis (Hidalgo-Castellanos et al., 2019). In order to test the potential role of Dat in the synthesis of these compounds, HPTLC was used to determine the PA profiles in bacteroids induced by the wild-type strain *Rlv* UPM791 and by the *dat*-deficient strain UPM1458 in pea and lentil. The results obtained in this analysis revealed that *Rlv* UPM791 pea bacteroids contained four PAs species (putrescine, norspermidine, spermidine and homospermidine, Figure 6A). The deletion of *dat* gene did not produce significant alterations of this PA profile. Analysis of extracts from lentil bacteroids showed a similar pattern. The PA profile of *Rlv* UPM791 vegetative cells was also analyzed. In this cell type only homospermidine and putrescine were found in the wild-type strain and, again, no differences were associated to the deletion of *dat* gene (Figure 6B). From these data it can be concluded that neither Dat protein nor the host legume affect PA profile of this rhizobial species.

In order to have a more comprehensive view of the potential metabolic role of Dat in symbiosis, a metabolomic approach was carried out using cell-free extracts from bacteroids induced by wild-type and *dat* deletion *Rlv* strains. An untargeted metabolome profiling of extracts from pea and lentil bacteroids induced by *Rlv* wild-type UPM791, by *dat*-mutant UPM1458 and by overexpressing UPM791(pBBRc189) strains was carried out using CE-ESI-MS-TOF, with six replicates per treatment. Relative abundance of metabolites and similarities between profiles were analyzed through heatmaps and hierarchical clustering, respectively. Seventy-one metabolites were identified in pea, and 58 compounds in the case of lentil.

**Figure 7:**
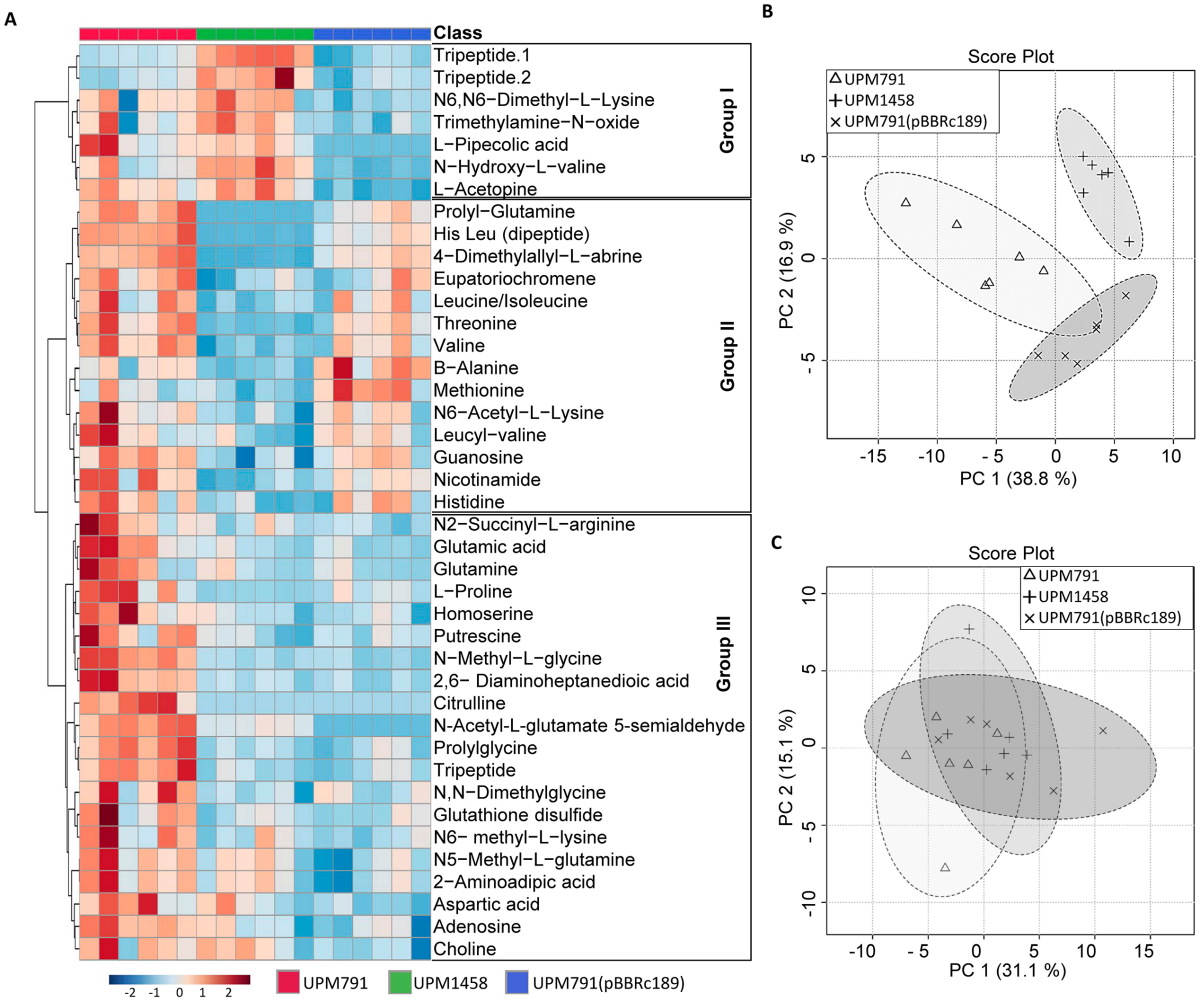
Metabolomic profile of lentil and pea bacteroids. **(A)** Heatmap of pea nodules inoculated with wild-type (UPM791), *dat* mutant (UPM1458) and *dat*-overexpression UPM791(pBBRc189) strains showing the 41 metabolites abundances on normalized scale with significant differences according to non-parametric Kruskal-Wallis test. Blue to red represent decreased to increased levels, respectively. The assay contained six replicates. **(B,C)** Principal Component Analysis (PCA) for all metabolites detected in pea and lentil bacteroids, respectively. Analyses were carried out with MetaboAnalyst5.0 (https://dev.metaboanalyst.ca/).

Sample clustering and Principal Component Analysis (PCA) showed a clear separation of metabolite profiles between strains in pea (Figure 7B) indicating that the presence of Dat caused a significant alteration of the cell metabolome. In the case of lentil, no clear separation of profiles could be found, which is consistent with the low expression of Dat in this host (Figure 7C and Supplementary Figure 3).

Heatmap analysis of the 41 compounds showing significant differences in pea bacteroids revealed three groups of compounds according to the pattern of variations across the strains tested (Figure 7A and Supplementary Table 3).

Group I corresponds to 7 compounds whose synthesis is apparently enhanced in the absence of Dat. The group includes tripeptides and non-proteinogenic amino acids (pipecolic acid, L-acetopine) whose role in the cell are not established.

Group II includes 14 compounds more abundant in the presence of Dat protein, and contains dipeptides, amino acids and amino acid derivatives. Some of the compounds in this group, such as β-alanine, threonine and methionine, can be mechanistically linked to aspartate semialdehyde, the substrate for Dat. The profile of β-alanine is quite interesting, as it has been shown as a precursor of pantothenate, and this has been further studied below. The potential role of other compounds showing consistent variations, such as dimethyl-allyl L-abrine, remains obscure.

The significance of Dat protein for 20 components of Group III is uncertain, since there is no clear correlation between overexpression of the gene and level of the compound. The group includes relevant species such as glutamate and homoserine, whose levels were reduced in the *dat*-deficient mutant vs. the wild-type strain, although the wide variations in the levels of these compounds in pea bacteroids make interpretation of the difference difficult. A clear explanation for these variations has not been found. This might not be a direct effect of the suppression of the DABA-AT activity in the mutant. Since such suppression should conserve both glutamate and homoserine, some kind of compensating mechanisms might be operating.

**Figure 8:**
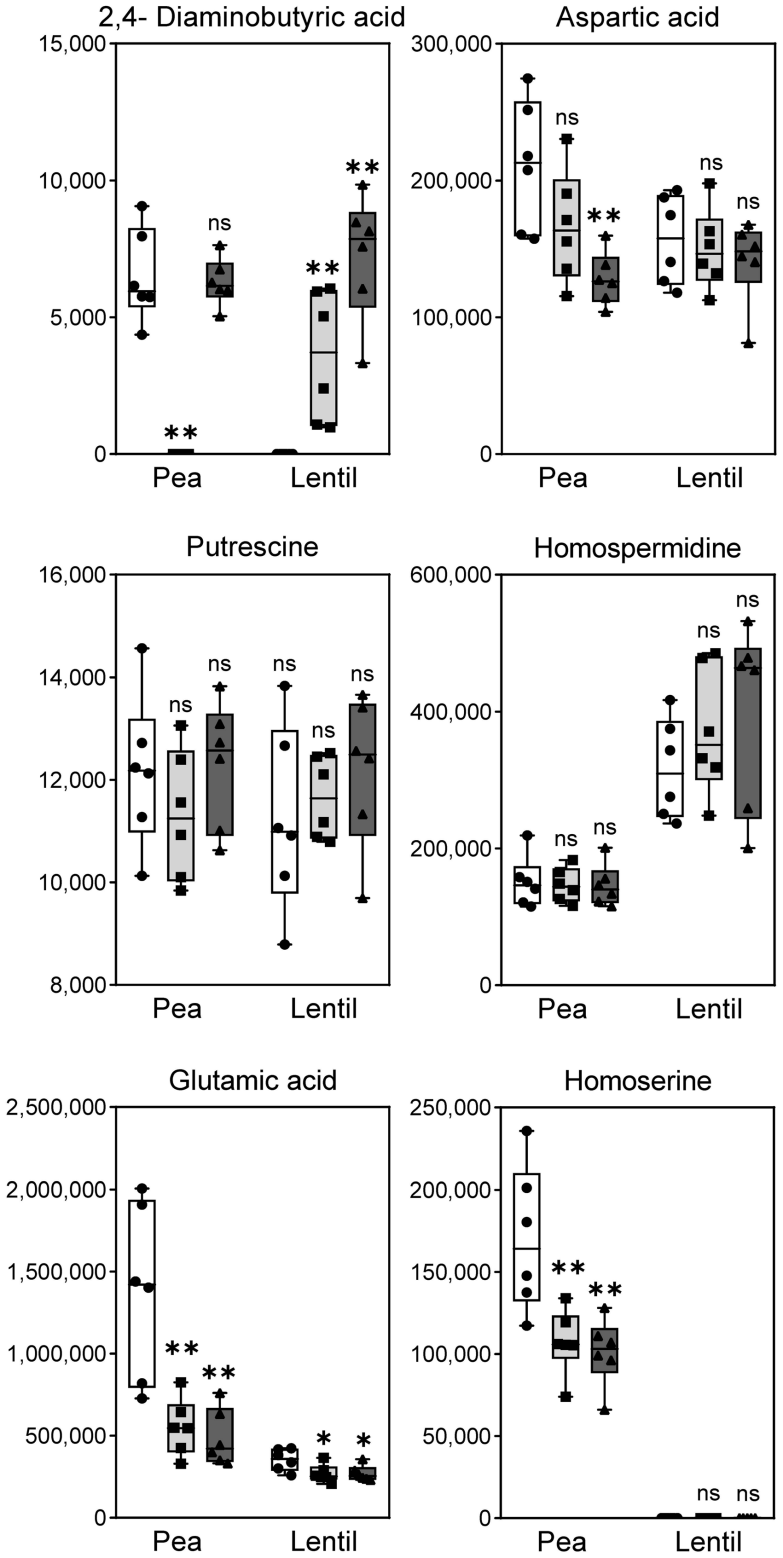
Targeted metabolomic analysis of metabolites in lentil and pea bacteroids. The graphic shows the levels of the indicated metabolites in bacteroids obtained from the indicated hosts. Strains: white bars, wild-type UPM791; light grey, *dat* mutant UPM1458; and dark grey, *dat*-overexpression strain UPM791(pBBRc189). Differences between wild-type and the other strains were analyzed by non-parametric Kruskal-Wallis.**P < 0.01, *P < 0.05; ns, indicates no statistically significant difference.

The untargeted metabolomic analysis did not identify DABA in the profile, so a more sensitive targeted analysis was carried out specifically searching for this compound. In this analysis, homospermidine, putrescine, glutamate, homoserine and aspartic acid, previously detected in the untargeted analysis, were also included as controls. The results obtained in this analysis (Figure 8 and Supplementary Table 4) revealed the presence of significant levels of diaminobutyrate in pea bacteroids induced by the wild-type strain but, interestingly, this compound was not detected in the *dat* mutant. DABA was detected also in pea bacteroids with extra copies of *dat* genes. These data strongly suggest that the direction of DABA-AT reaction in pea nodules is toward the synthesis of diaminobutyrate from L-Asa. A different situation was observed in the case of lentil bacteroids, in which extracts from the wild-type had no detectable levels of DABA. In contrast, this compound was present in the mutant, suggesting that DABA is consumed by Dat protein in this legume host (Figure 8 and Supplementary Table 5). Although detected at levels *ca.* 10 times higher as consequence of the improved sensitivity of targeted analysis, the levels of homospermidine, putrescine, glutamate, homoserine and aspartic acid followed a pattern similar to that observed in the untargeted analysis (Supplementary Tables 3, 4).

### Growth with homoserine as N substrate is altered in the *Rlv dat* mutant

**Figure 9:**
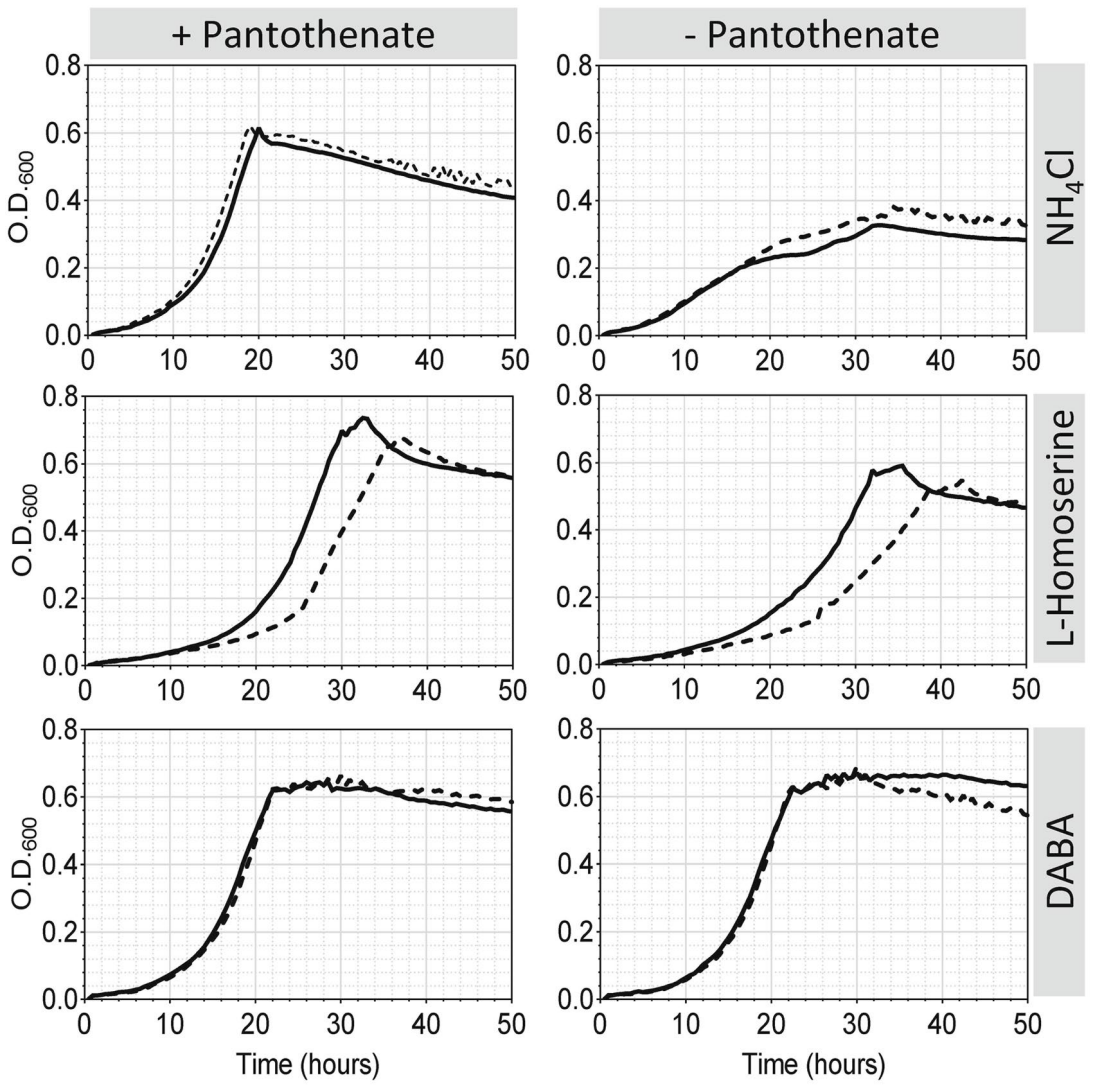
Effect of *Rlv* UPM791 *dat* mutation and N source on growth and pantothenate auxotrophy. Cells were grown in UMS containing ammonium chloride, L-homoserine or DABA as nitrogen sources (10 mM). Media were prepared either with pantothenate (8.4 μM) (left panels) or without pantothenate (right panels). Growth curves correspond to the mean of four replicate cultures of *Rlv* UPM791 (solid line) or its *dat-*mutant derivative *Rlv* UPM1458 (dashed line). All standard deviations were below 6.5%.

Since the metabolomic analysis of pea bacteroids revealed that (i) Dat protein is likely involved in the generation of diaminobutyrate in the cell; and (ii) the presence of homoserine in the bacteroid, it can be concluded that this protein might be involved in the conversion of L-Asa into diaminobutyrate in pea bacteroids, with L-Asa likely originating from homoserine via homoserine dehydrogenase (HSD). To test this hypothesis, the effect of *dat* deletion on the growth of *Rlv* UPM791 was analyzed using either ammonium or L-homoserine as N substrate. Cultures from both wild-type and *dat*-deleted mutant grew equally well in UMS minimal medium with glucose/ammonium as C/N sources (Figure 9, left upper panel). However, when the same medium was modified to include L-homoserine as N source, the mutant strain showed a clear impairment in growth (Figure 9, middle left). The suitability of DABA, product of the Dat protein, as nitrogen source was also tested. In this case, no difference was found in the growth curves of wild-type and mutant strains (Figure 9, bottom left).

### Homoserine and DABA overcome pantothenate auxotrophy in *Rlv* UPM791

An effect of the mutation of *dat* on growth on L-homoserine has been shown. Also, a reduction of the level of β-alanine, a precursor of pantothenate, was observed in the metabolomic analysis of the *dat* mutant. Given the role of Dat in the synthesis of β-alanine described in other bacteria (Perchat et al., 2022), we hypothesized that homoserine and DABA could be used as sources of pantothenate in *Rlv UPM*791, and that Dat protein might be involved in this. To test this hypothesis, the growth of wild-type and *dat*-deficient strains in modified pantothenate-lacking UMS media containing each of the N sources cited above were compared. When NH_4_ was the N source (standard UMS medium), the lack of pantothenate led to a clear decrease of the growth (Figure 9, upper right panel), thus confirming the auxotrophy of *Rlv* for pantothenate in standard mineral medium. Interestingly, when DABA was used as nitrogen source (Figure 9 bottom right panel), pantothenate was not required, indicating that DABA is able to supply all required pantothenate. Finally, in the presence of homoserine as N source there was a small but significant decrease in growth in the absence of pantothenate (Figure 9 panel medium right), indicating that this compound was able to partially supply the pantothenate required by the cell.

**Figure 10:**
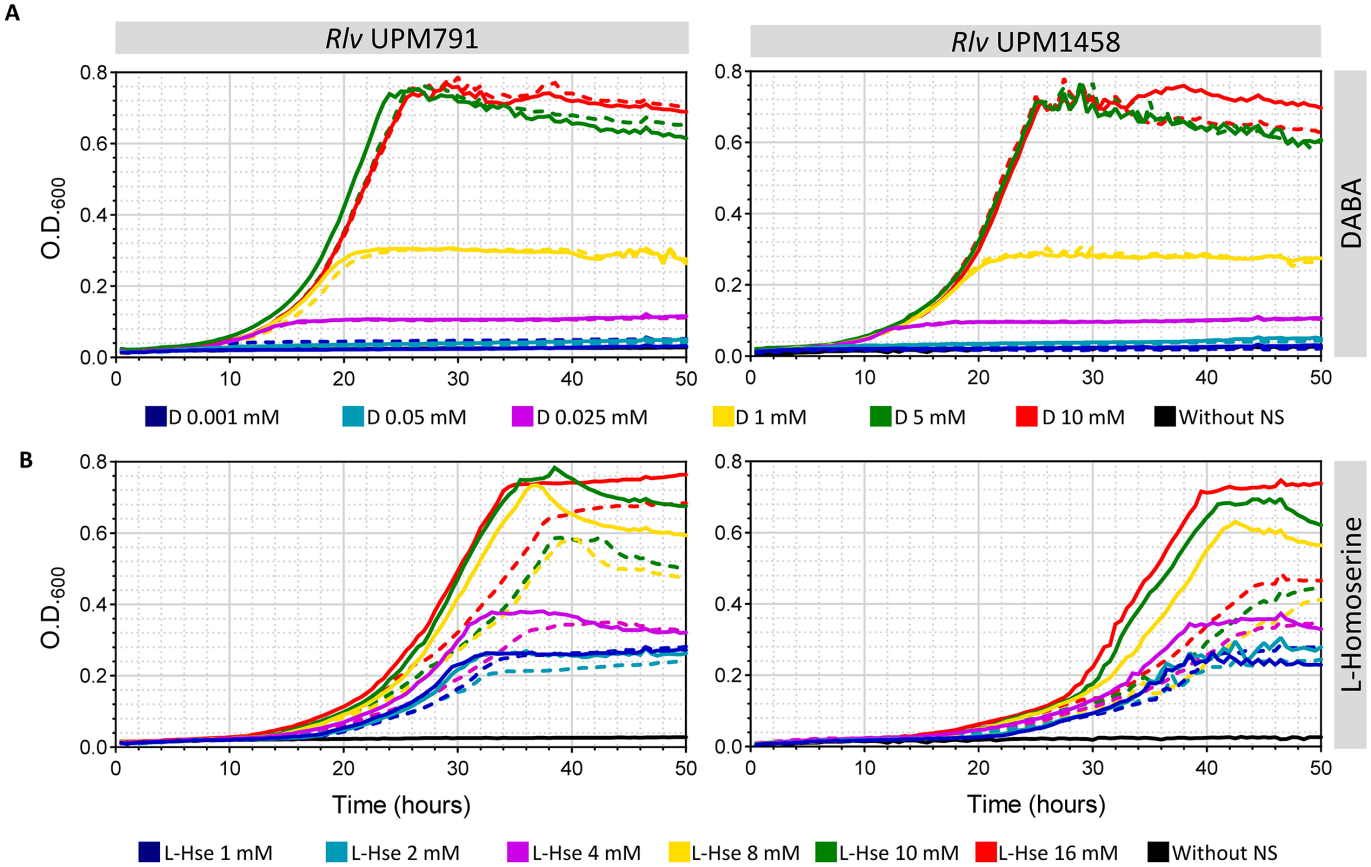
Effect of L-homoserine and DABA in growth and pantothenate auxotrophy of *Rlv* UPM791. Growth curves correspond to *Rlv* strains UPM791 (wild-type) and UPM1458 (*dat^−^*) grown in UMS medium modified by incorporating the indicated amounts of DABA **(A)** or L-homoserine **(B)** as only nitrogen sources. Media were prepared either without pantothenate (dashed lines) or supplemented with 8.4 μM pantothenate. Points represent the mean of three replicates. All standard deviations were below 8.1%.

In order to further analyze the effect of these two compounds in pantothenate auxotrophy, media with increasing amounts of either DABA or L-homoserine as the only N source were used both in the presence and in the absence of pantothenate (Figure 10). In the case of DABA, there were no differences associated to the presence of pantothenate at any DABA concentration (Figure 10A), indicating that this compound was able to supply all the required pantothenate to both wild type and mutant strains. Under these conditions, the growth was limited only by the amount of N supplied by DABA up to *ca.* 5 mM.

Growth curves of both wild-type and mutant strains with increasing amounts of L-homoserine as the only N source revealed that this compound is not as efficient as DABA in providing pantothenate, but still provides substantial growth in the absence of pantothenate for the wild-type strain (Figure 10B). In this case, however, some increments associated to the addition of pantothenate were observed, accounting for a fraction of the total growth with pantothenate. And, interestingly, the difference between the growth without pantothenate (dashed lines) and with pantothenate (solid lines) was clearly bigger in the case of the mutant. These data support the idea that the mutation does interfere with the ability of L-homoserine to provide pantothenate, likely via the production of less DABA.

## Discussion

In this work *Rlv* UPM791 Dat is shown as a diaminobutyrate 2-oxoglutarate aminotransferase (E.C. 2.6.1.76) that contributes to the growth with homoserine as nitrogen source, and to optimal levels of symbiotic performance and nodulation competitiveness in pea, but not in lentil. This protein was identified in an extensive search for host-dependent proteins with the final aim to find traits involved in bacterial adaptation to specific legume hosts (Durán et al., 2021). Purified, active Dat enzyme showed an oligomeric structure, likely a tetramer, similarly to what has been described for other enzymes of this class (Ikai and Yamamoto, 1997). The available data indicate that the enzyme operates in pea bacteroids toward the production of diaminobutyrate from aspartate semialdehyde and glutamate.

Regarding potential roles of Dat in *Rhizobium* metabolism, its involvement of processes such as synthesis of ectoine (Schwibbert et al., 2011) and siderophores (Lynch et al., 2001) was discarded on the basis of the lack of other genes required for these pathways. Analysis of PA profiles revealed the presence of putrescine, norspermidine, spermidine and homospermidine, homospermidine being the most prominent species. These data were confirmed in the metabolomic analysis (Supplementary Table 3), with the exception that the norspermidine deduced from the HPTLC probably corresponds to N-acetyl-norspermidine, according to metabolomics data. Putrescine, spermidine and homospermidine were previously described in alfalfa bacteroids of *S. meliloti* (Becerra-Rivera et al., 2020). The profile observed in free-living cells coincides with that described previously for *Rlv* 3841 (Becerra-Rivera et al., 2020). Regarding the potential effect of *Rlv* Dat on the synthesis of PAs, the conservation of polyamine profiles in the *wt/dat*^−^ comparison indicate that polyamine synthesis is not dependent on Dat enzyme in this bacterium.

The available evidence indicate that Dat is involved in homoserine metabolism, as the *dat* mutant is less efficient in using L-Hse as nitrogen source. Metabolomic analysis revealed that a major difference in extracts from bacteroids induced by *Rlv* UPM791 in pea and lentil nodules is the presence of L-Hse. L-homoserine is a known component of pea root exudates (Rovira, 1956) also described in pea bacteroids, which likely obtain it from the plant (Terpolilli et al., 2016). Growth curves shown in this work indicate that L-Hse is a N source less efficient than ammonium for *Rlv* UPM791. In a medium with L-Hse the growth difference between wild-type and *dat* mutant is not dramatic. However, a stronger phenotype regarding growth was not expected on L-Hse, as the bacterium contains a full gene cluster (RLV_473–482) for homoserine catabolism in a different genome localization (Vanderlinde et al., 2014).

The substrate for Dat, L-Asa, can be generated in the cell by homoserine dehydrogenase (HSD). Interestingly, *Rlv* UPM791 encodes two HSDs (RLV_0467 and RLV_4488) showing very distant relationship (26.9% identity). One of the proteins (RLV_0467) belongs to a novel family of HSDs recently described in *Arthrobacter nicotinovorans* and showing a strong unidirectionality toward oxidation of L-Hse into L-Asa (Liang et al., 2022). These authors hypothesized that members of this novel group of HSDs evolved in homoserine-rich habitats to effectively use this compound as a source of carbon and/or nitrogen, a situation likely to occur in pea rhizosphere. Furthermore, it has to be noted that: (i) RLV_467 is encoded in a region close to the gene cluster involved in L-Hse catabolism (Vanderlinde et al., 2014); (ii) this protein was detected in *Rlv* UPM791 pea bacteroids (Durán et al., 2021); and (iii) its ortholog in *Rlv* 3841 (pRL80071) was highly induced in pea rhizosphere (Ramachandran et al., 2011). All these data suggest a role as an L-homoserine-consuming HSD expressed during the interaction with pea plants. The other HSD present in *Rlv* genome (RLV_4488) is related to HSDs that catalyze the reversible conversion of L-Asa into L-Hse in the aspartate pathway, usually displaced toward synthesis of L-Hse. The gene is located in the *Rlv* UPM791 chromosome, and the corresponding protein was present in extracts from vegetative cells and pea and lentil bacteroids (Durán et al., 2021). Furthermore, its ortholog in *Rlv* 3841 (RL2097) was described as essential for growth in mineral media and also for fitness of the bacterium in the rhizosphere and in symbiosis with pea (Wheatley et al., 2020). This particular enzyme is likely involved in making L-Hse, a precursor for the synthesis of essential amino acids such as L-threonine, L-methionine, and L-isoleucine in the L-aspartate family amino acids.

The available data suggest that metabolism of *Rlv* UPM791 pea bacteroids is adapted to the presence of L-Hse as substrate, and it can be hypothesized that part of this substrate is converted into DABA via Dat. DABA has been recently shown as an intermediate for pantothenate synthesis through its involvement in the generation of β-alanine in *Acinetobacter baylyi* (Perchat et al., 2022). Pantothenate is a precursor of CoA, a key compound for cells, particularly for those relying in tricarboxylic acid cycle. Many α-*Proteobacteria,* including *Rlv* UPM791*,* lack aspartate decarboxylase (PanD; López-Sámano et al., 2020), the major enzyme involved in the direct conversion of aspartate into β-alanine. Thus, these bacteria require an alternative route or the presence of pantothenate in the medium. In particular, *Rlv* is considered a pantothenate auxotroph, and standard recipes for mineral media defined for this species (Rmin, UMS) include this vitamin (O’Gara and Shanmugam, 1976; Wheatley et al., 2017). Metabolomic analysis carried out in this work showed that *dat* mutation results in a significant reduction of β-alanine in pea bacteroids. Interestingly, the presence of DABA in lentil bacteroids induced by the *dat* mutant was accompanied by a significant increase in the level of β-alanine (Supplementary Table 3). Furthermore, the addition of DABA to the medium overcame pantothenate auxotrophy of free-living cells of *Rlv* UPM791. The same was also observed, although with lower efficiency, when using homoserine as N source. All these data indicate that Dat-dependent DABA is involved in the synthesis of pantothenate via β-alanine. There are not many data on alternative routes for pantothenate synthesis in rhizobia. In *Rhizobium etli* CFN42, a β-alanine synthase involved in pyrimidine degradation was able to generate β-alanine using carbamoyl-β-alanine as substrate (López-Sámano et al., 2020). The ability to synthesize pantothenate derived from homoserine might represent an advantage in habitats in which this compound is abundant, as it is the case in the rhizosphere and nodules of pea plants. It is interesting to note that pantothenate synthesis is essential in other host–microbe interactions, as it is the case for chronic infection in the cellular parasite *Toxoplasma gondii* (Lunghi et al., 2022).

The impaired symbiotic performance associated with the *dat* mutant is not likely due to a direct effect on the activity of nitrogenase, as the mutation did not affect the level of acetylene reduction activity. The presence of L-homoserine in the bacteroids, and the role of Dat enzyme in the growth based on this compound and in pantothenate synthesis, suggest that a better use of homoserine and a more abundant provision of pantothenate could be responsible for the observed decrease of biomass accumulation in the mutant strain. The high levels of homoserine in pea seedlings and pea rhizosphere (Rovira, 1956; Rozan et al., 2001) is a likely reason explaining the reduced competitiveness of the *dat*-mutant. A similar phenotype was described for *Rlv* mutants deficient in the homoserine degradation cluster (Vanderlinde et al., 2014).

The regulatory mechanism explaining the differential expression of *dat* in pea vs. lentil bacteroids has not been elucidated here. Reporter gene analyses revealed high levels of expression of *dat* gene in the nodule region containing mature bacteroids in pea, but not in lentil. A clear explanation for the atypical expression of the gene in the meristematic area of lentil nodules has not been found. Both hosts induce functional, nitrogen fixing nodules when inoculated with *Rlv* UPM791. Previous studies on host-dependent expression of another enzyme of the same strain (hydrogenase) had revealed that the control was exerted at transcriptional level (Brito et al., 2008), as it has been observed here. A pea-specific GntR-type transcriptional regulator whose mutation resulted in impaired symbiotic performance was identified in the previous proteomic analysis (Durán et al., 2021). However, analysis of a mutant in this regulator has shown that there is not a significant effect of this transcriptional regulator in *dat* expression (data not shown). Also, a mutation in the lentil-specific repressor encoded in chrUPM791_04389 did not affect expression of the gene. No evident binding sites for known symbiotic transcriptional regulators (NifA, FnrN) were found in the region upstream of the gene. Furthermore, analyses with the reporter gene fusion in vegetative cell cultures grown in the presence of homoserine and/or a known symbiotic condition (microaerobiosis) did not show significant activation of *dat* promoter activity (data not shown). It is possible that a combination of factors, some symbiotic ones combined with the presence of L-homoserine, is required for the activation of *dat* gene.

In conclusion, the data presented here indicate that *Rlv* UPM791 Dat enzyme is part of an adaptation mechanism of this bacterium to a homoserine-rich environment such as pea nodule and rhizosphere. This enzyme can be considered a potential tool in the development of more efficient inoculants for this agriculturally relevant legume species.

## Data availability statement

The original contributions presented in the study are included in the article/Supplementary material, further inquiries can be directed to the corresponding author.

## Author contributions

MB-G: formal analysis, investigation, methodology, data curation, visualization, and writing—original draft and review. MA: conceptualization, methodology, project administration, supervision, validation, and writing—original draft and review. CB and ÁL-G: metabolomic data acquisition and analysis. MD: methodology and supervision concerning to PAs analysis and validation writing—original draft. JP: conceptualization, methodology, project administration, funding acquisition, supervision, and writing—original draft and review. All authors contributed to the article and approved the submitted version.

## Funding

This work was funded by a grant from Agencia Española de Investigacion, Ministerio de Ciencia e Innovación (PID2021-124344OB-I00). MB-G was recipient of a fellowship from Programa Propio from Universidad Politécnica de Madrid.

## Conflict of interest

The authors declare that the research was conducted in the absence of any commercial or financial relationships that could be construed as a potential conflict of interest.

## Publisher’s note

All claims expressed in this article are solely those of the authors and do not necessarily represent those of their affiliated organizations, or those of the publisher, the editors and the reviewers. Any product that may be evaluated in this article, or claim that may be made by its manufacturer, is not guaranteed or endorsed by the publisher.

## Supplementary material

The Supplementary material for this article can be found online at: https://www.frontiersin.org/articles/10.3389/fmicb.2023.1182563/full#supplementary-material

## Footnotes

^1^ https://dev.metaboanalyst.ca/
