# Supplementary material for: A host-specific diaminobutyrate aminotransferase contributes to symbiotic performance, homoserine metabolism, and competitiveness in the Rhizobium leguminosarum/Pisum sativum system
Source: Front Microbiol. 2023 May 16;14:1182563. doi: 10.3389/fmicb.2023.1182563 (PMC10228743; doi:10.3389/fmicb.2023.1182563)
Supplement: Supplementary file 1 [file Data_Sheet_1.pdf]

Supplementary Material

A host-specific diaminobutyrate aminotransferase contributes to symbiotic performance, homoserine metabolism, and competitiveness in the *Rhizobium. leguminosarum* / *Pisum sativum* system.

Marta Ballesteros-Gutiérrez[1], Marta Albareda[1,2], Coral Barbas[3], Ángeles López-Gonzálvez[3], Michael F. Dunn[4], y José M. Palacios[1,2*.]

[1]Centro de Biotecnología y Genómica de Plantas (CBGP, UPM-INIA/CSIC), Universidad Politécnica de Madrid, Instituto Nacional de Investigación y Tecnología Agraria y Alimentaria, Consejo Superior de Investigaciones Científicas, Campus de Montegancedo, Pozuelo de Alarcón, Spain

[2]Departamento de Biotecnología-Biología Vegetal, Escuela Técnica Superior de Ingeniería Agronómica, Alimentaria y de Biosistemas, Universidad Politécnica de Madrid, Madrid, Spain

[3]Center for Metabolomics and Bioanalysis (CEMBIO), Facultad de Farmacia, Universidad San Pablo-CEU, CEU Universities, Boadilla del Monte, Spain

[4]Programa de Genómica Funcional de Procariotes, Centro de Ciencias Genómicas-Universidad Nacional Autónoma de México, Cuernavaca, Mexico

*** Correspondence:** José Manuel Palacios. Jose.palacios@upm.es

ORCID

Marta Ballesteros-Gutiérrez: https://orcid.org/0000-0002-8995-1739
Marta Albareda: https://orcid.org/0000-0002-4343-4445
Coral Barbas: https://orcid.org/0000-0003-4722-491X
Ángeles López-Gonzálvez: https://orcid.org/0000-0002-6363-7135
Michael F. Dunn: https://orcid.org/0000-0002-8657-0375
José M. Palacios: https://orcid.org/0000-0002-2541-8812

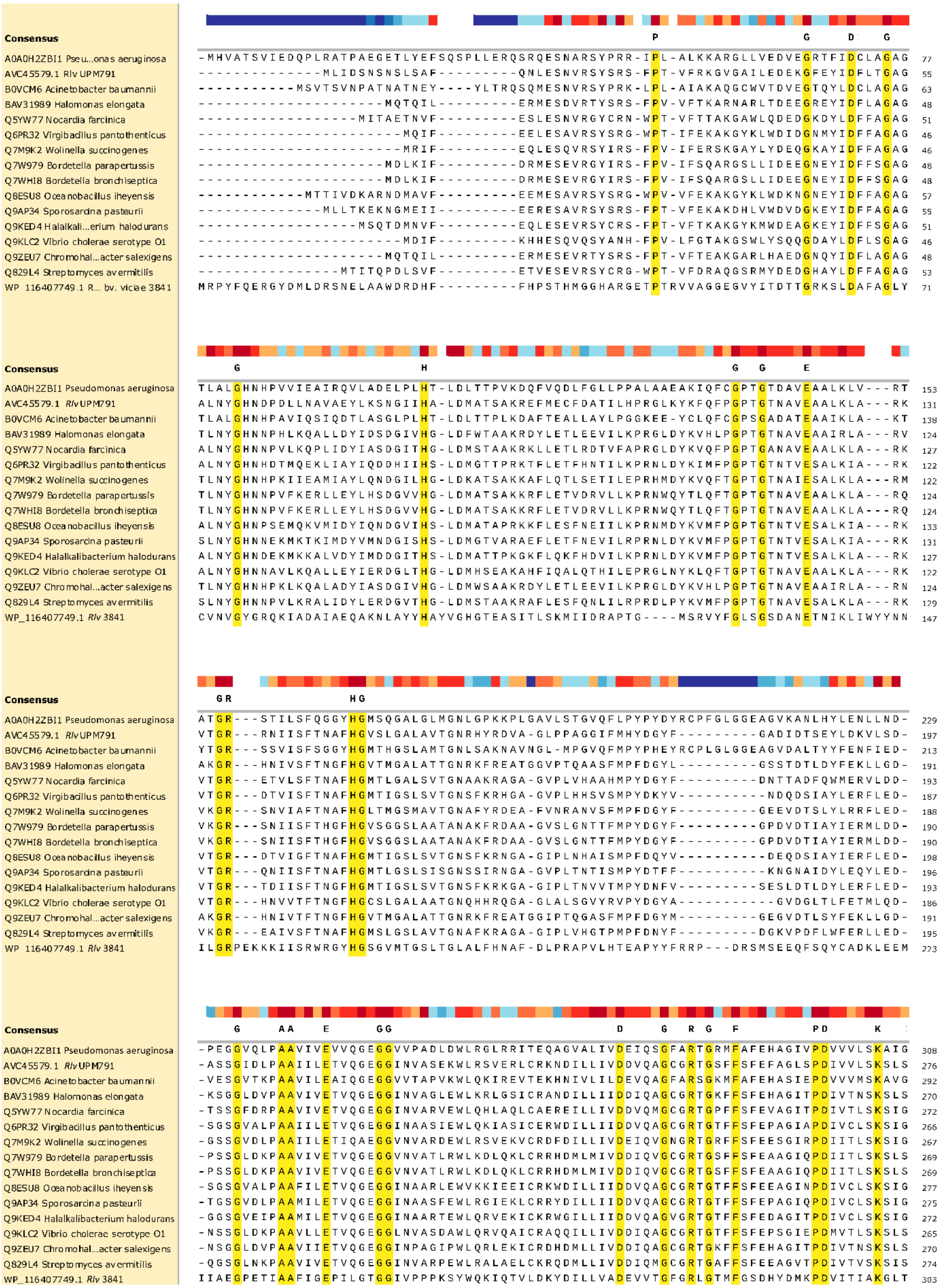

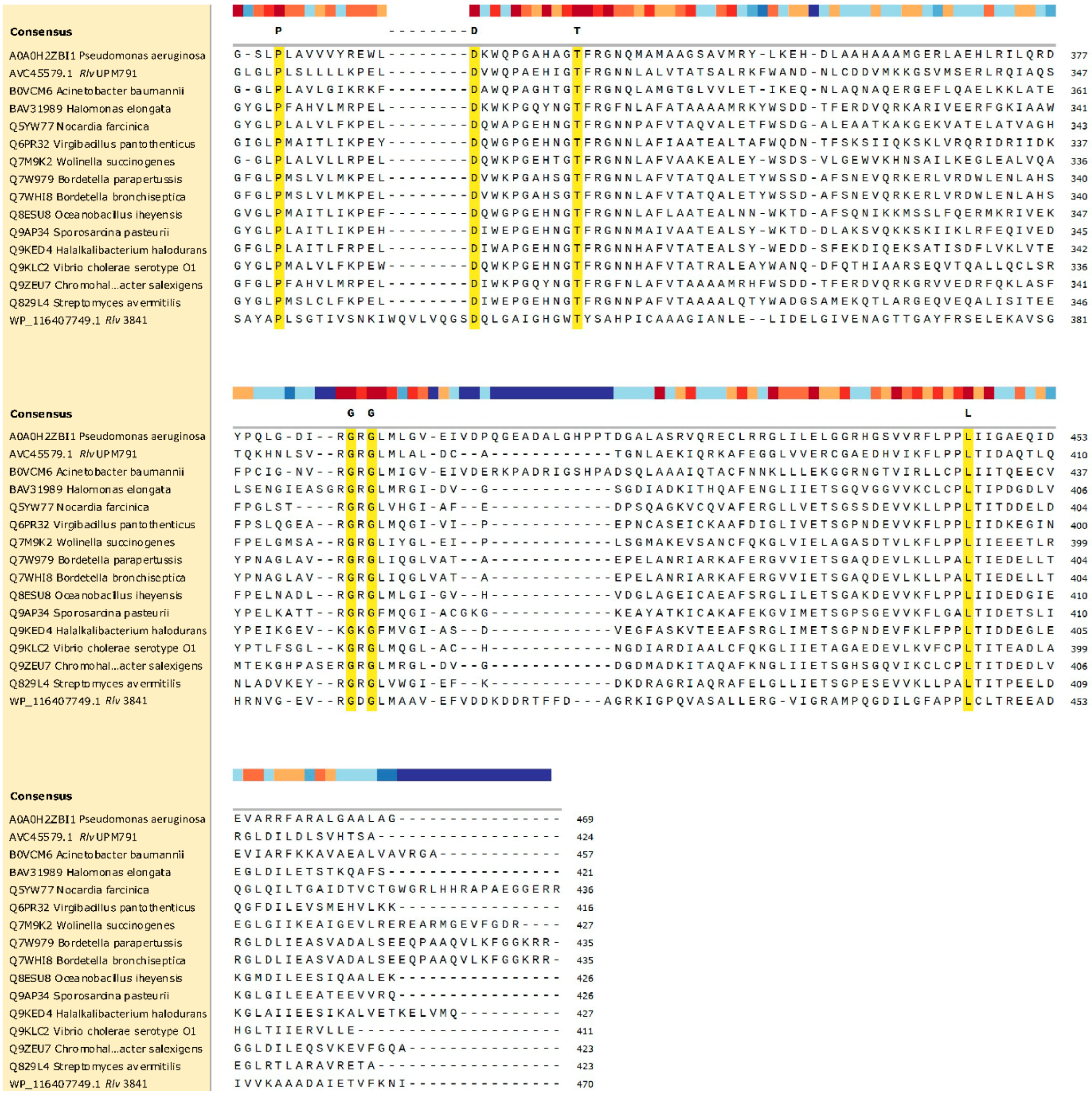

Supplementary Figure 1. Multiple sequence alignment of DABA-AT proteins. Uniprot/NCBI accession codes are followed by the scientific name of the corresponding microorganism. The multiple sequence alignment was created with MUSCLE (Edgar, 2004), and visualized with SnapGene (www.snapgene.com). The areas shaded in yellow highlight the fully conserved residues, while the bar over the alignment illustrate the degree of conservation (deep blue, no conservation, red = high degree of conservation).

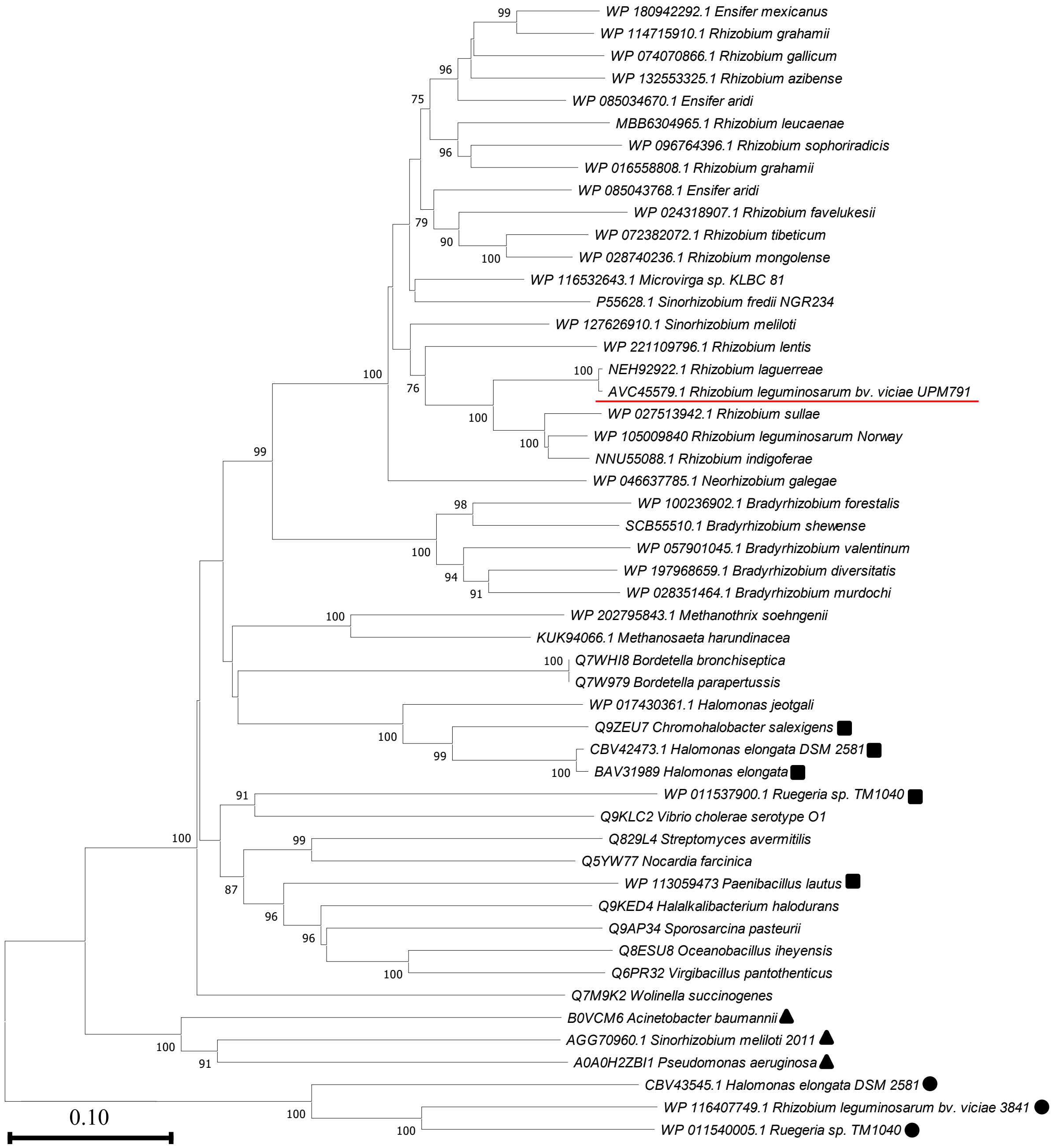

Supplementary Figure 2. Phylogenetic tree of DABA-AT proteins from different prokaryotes. A total of 50 sequences were analyzed using the Neighbor-Joining method (Saitou and Nei, 1987). The phylogenetic distances were computed using the p-distance method (Nei and Kumar, 2000). Only bootstrap values greater than 75% (calculated for 1000 subsets) are shown (Felsenstein,1985). Dat protein of *Rlv* UPM791 is underlined. DABA-AT proteins marked with squares (■) are involved in ectoine biosynthesis; the ones with circles (●) in ectoine degradation, and those with triangles (▲) in synthesis of siderophores or polyamines. Accession numbers from GenBank are followed by organism's name and strain. The scale bar indicates number of substitutions per site. Phylogenetic analysis was conducted with MEGA11 (Tamura et al., 2021).

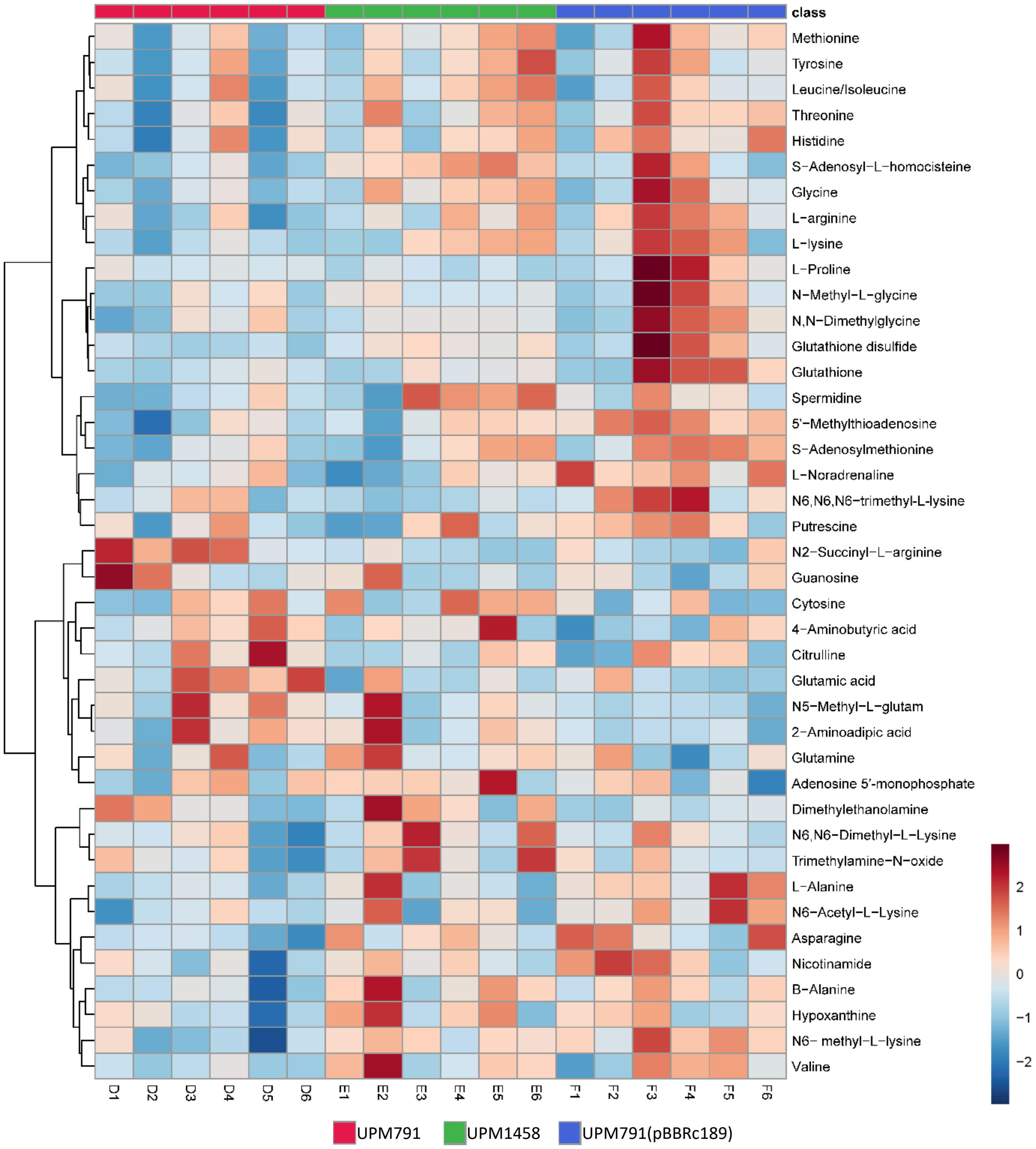

Supplementary Figure 3. Heat-map of bacteroids of lentil nodules of untarget analysis. Plants were inoculated with wild-type (UPM791), *dat* mutant (UPM1458) and *dat*-overexpression [(UPM791(pBBRc189)] strains showing metabolites abundances on normalized scale. Blue to red represent decreased to increased levels, respectively. The samples are composed by six replicates.

Supplementary Table 1. Bacterial strains and plasmids used in this work.

Strain or plasmid	Genotype or relevant characteristic(s)	Source or reference
Escherichia coli		
DH5α	*recA1 endA1 gyrA96 thi hsdRl7 supE44 relA1* Δ(*lacZYA-argF)U169* (φ80*lacZ*ΔM15) *deoR phoA*	(Hanahan, 1983)
GM119	*dam-3 dcm-6 metBI gaIK2 gaIT22 lacY tsx-78 supE44 (thi-1) tonA3I mtl-I)*	(Arraj and Marinus, 1983)
S17.1	*thi pro hsdR hsdM*$^{+}$ *recA* RP4::2-Tc::Mu-Kan::T7; Sp^{r} Sm^{r}	(Simon et al., 1983)
Rhizobium leguminosarum **bv. viciae**		
UPM791	128C53 wild type; Str^{r} Nod^{+} Fix^{+} Hup^{+}	(Leyva et al., 1987)
UPM1458	UPM791 *Δc189*::ΩSp*ec*, Sp^{r}	This work
**Plasmids**		
pCR2.1-TOPO	PCR product cloning vector; Amp^{r}, Km^{r}	Invitrogen
pBlueScript-II KS^{+}	PCR product cloning vector; Amp^{r}	Stratagene
pTPc189	pCR2.1-TOPO containing *c189* construction for deletion of UPM791 *c189*, Km^{r}, Sp^{r}	This work
pK18*mobsacB*	Integrative vector pUC18 derivative; *lacZ mob* site *sacB,* Km^{r}	(Schäfer et al., 1994)
p*K*c189	pK18*mobsacB* carrying *c189* construction for deletion of *c189,* Km^{r}, Sp^{r}	This work
pBluecomc189	pBlueScript containing *c189* with native promoter, Amp^{r}	This work
pBluetauc189	pBlueScript containing *c189* gene fused to a *Strep*-tag II coding sequence in its 3'-end and *c189* promoter, Amp^{r}	This work
pBBR1MCS-5	Broad-host-range vector with a Plac promoter, Gm^{r}	(Kovach et al., 1995)
pBBRc189	pBBR1MCS-5 containing *c189* and its native promoter, Gm^{r}	This work
pLMB51	*gusA* delator gen vector with taurine dependent promotor, Tc^{r}	(Tett et al., 2012)
pLMBc189$_{ST}$	pLMB51 containing *c189* gene fused to a *Strep-tag* II coding sequence in its 3'-end and *c189* promoter, Tc^{r}	This work
pHP45Ω	Sp^{r} ,Amp^{r}, Sm^{r}	(Fellay et al., 1987)

Supplementary Table 2. Oligonucleotides used in this work.

Primers	Sequence (5'- 3')	Description
**DABA_AT_P1**	CGTTGTGCCGAAAGTGCTTG	Generation of Δ*c189* (UPM1458) mutant
**DABA_AT_P2**	*GGGCCCGGG*GCAGAAAGCGAGTTGGAGTTG	
**DABA_AT_P3**	*CCCCCCCCC*GTTTCTTCCACCCCTGACGA	
**DABA_AT_P4**	GCAACGCTCAACAAAGACCA	
**spcR_P5_DABA_AT**	*CCCGGGCCC*GATCCTTGGAGCCCTTGC	pHP45Ω spectinomycin resistance cassette
**spcR_P6_DABA_AT**	*GGGGGGGGG*AGATTTTAATGCGGATGT	
**DABA_AT_COM_F**	AAATCTAGACGCAAGGCCACGCAGCCTGCTGAAT	Complementation and over-expression
**DABA_AT _COM_R**	AAAAAGCTTTCAAGCGCTTGTGTGGACAGACAG	
**DABA _PLMB51_F**	AATGGATCCTACGGGCCTCGATTACGTTG	$C189_{ST}$ taurine-inducible system
**DABA _PLMB51_strep_R**	ATTTCTAGA**TCACTTTTCGAACTGCGGGTGGCTCCA GCTAGC**AGCGCTTGTGTGGACAGACAGGTCCAGAAT	
**M13_R_TOPO**	CAGGAAACAGCTATGAC	Primers used for checking the constructions
**M13_F_TOPO**	GTAAAACGACGGCCAGT	
**pLMB51_Check_F**	GTTTCTACAGGACGGACCAT	
**pLMB51_Check_R**	AAGCGGGGCGACATAACCAA	
**c189_qPCR_F**	GATCCCGATTTGCTCAACGC	RT-qPCR
**c189_qPCR_R**	ACGTGGGTGCAAAATTGTCG	
**rpoD_qPCR_F**	GCTTCGACCATTTCCTTCTTGG	
**rpoD_qPCR_R**	GATGAAGTCGATCGGAATCTG	
**gabT_qPCR_F**	ACGCGATCAACCTTACGGA	
**gabT_qPCR_R**	GGCAAAATCGATGTAGCGG	

Nucleotides underline are restriction sites for cloning, detailed in material and methods part. Nucleotides in bold form the sequence for *StrepTaq II*.

Supplementary Table 4. Targeted analysis of pea bacteroid metabolites.

Compound	Means of 6 replicates			% of change B vs A[a]	% of change C vs A[b]	% of change C vs B[c]	p-kruskal B vs A[a]	p-kruskal C vs A[b]	p-kruskal C vs B[c]
	UPM791	UPM1458	UPM791 (pBBR-c189)						
Putrescine	12174.1	11312.9	12284.2	-7.1	0.9	8.6	0.394	0.699	0.180
2,4-diamonobutyric acid	6506.1	-	6286.8	**-100.0**	-3.4	-	**0.002**	0.937	**0.002**
Aspartic acid	211659.1	166486.8	128249.6	-21.3	**-39.4**	-23.0	0.132	**0.004**	0.093
Glutamic acid	1383139.7	553019.1	486722.9	**-60.0**	**-64.8**	-12.0	**0.009**	**0.004**	0.589
Homospermidine	150968.4	146443.1	146020.8	-3.0	-3.3	-0.3	1.000	0.937	0.818
Homoserine	169946.4	107414.2	101300.1	-36.8	**-40.4**	**-5.7**	**0.009**	**0.004**	0.699

[a] Comparison between UPM1458 (B) vs UPM791 (A).
[c] Comparison between UPM791(pBBRc189) (C) vs UPM791 (A).
[b] Comparison between UPM791(pBBRc189) (C) vs UPM1458 (B).
Data in bold shows significant differences.

Supplementary Table 5. Targeted analysis of lentil bacteroid metabolites.

Compound	Means of 6 replicates			% of change B vs A[a]	% of change C vs A[b]	% of change C vs B[c]	p-kruskal B vs A[a]	p-kruskal C vs A[b]	p-kruskal C vs B[c]
	UPM791	UPM1458	UPM791 (pBBR-c189)						
Putrescine	11232.7	11656.0	12180.3	3.8	8.4	4.5	0.818	0.485	0.310
2,4-diamonobutyric acid	0	3583.3	7239.8	-	-	**102.0**	***0.002***	***0.002***	*0.026*
Aspartic acid	156766.1	149759.7	141139.6	-4.5	-10.0	-5.8	0.818	0.699	1.000
Glutamic acid	353148.8	269892.3	270863.0	**-23.6**	**-23.3**	0.4	**0.026**	**0.041**	0.818
Homospermidine	316332.9	372286.5	399729.8	17.7	26.4	7.4	0.485	0.240	0.818
Homoserine	0	**0**	0	-	-	-	-	-	-

[a] Comparison between UPM1458 (B) vs UPM791 (A).
[c] Comparison between UPM791(pBBRc189) (C) vs UPM791 (A).
[b] Comparison between UPM791(pBBRc189) (C) vs UPM1458 (B).
Data in bold shows significant differences.

Bibliography

Arraj, J. A., and Marinus, M. G. (1983). Phenotypic reversal in dam mutants of *Escherichia coli* K-12 by a recombinant plasmid containing the *dam*$^{+}$ gene. *J. Bacteriol.* 153, 562–565. doi: 10.1128/jb.153.1.562-565.1983.

Edgar, R. C. (2004). MUSCLE: Multiple sequence alignment with high accuracy and high throughput. *Nucleic Acids Res.* 32, 1792–1797. doi: 10.1093/nar/gkh340.

Fellay, R., Frey, J., and Krisch, H. (1987). Interposon mutagenesis of soil and water bacteria: a family of DNA fragments designed for in vitro insertional mutagenesis of Gram-negative bacteria. *Gene* 52, 147–154. doi: 10.1016/0378-1119(87)90041-2.

Felsenstein, J. (1985). Confidence limits on phylogenies: an approach using the bootstrap. *Evolution* 39, 783–791. doi: 10.1111/j.1558-5646.1985.tb00420.x.

Hanahan, D. (1983). Studies on transformation of *Escherichia coli* with plasmids. *J. Mol. Biol.* 166, 557–580. doi: 10.1016/S0022-2836(83)80284-8.

Kovach, M. E., Elzer, P. H., Steven Hill, D., Robertson, G. T., Farris, M. A., Roop, R. M., et al. (1995). Four new derivatives of the broad-host-range cloning vector pBBR1MCS, carrying different antibiotic-resistance cassettes. *Gene* 166, 175–176. doi: 10.1016/0378-1119(95)00584-1.

Leyva, A., Palacios, J. M., and Ruiz-Argüeso, T. (1987). Conserved plasmid hydrogen-uptake (hup)-specific sequences within *Hup+ Rhizobium leguminosarum* strains. *Appl. Environ. Microbiol.* 53, 2539–2543. doi: 10.1128/aem.53.10.2539-2543.1987.

Nei, M., and Kumar, S. (2000). *Molecular Evolution and Phylogenetics*. New York: Oxford University Press.

Saitou, N., and Nei, M. (1987). The neighbor-joining method: a new method for reconstructing phylogenetic trees. *Mol. Biol. Evol.* 4, 406–425. doi: 10.1093/oxfordjournals.molbev.a040454.

Schäfer, A., Tauch, A., Jäger, W., Kalinowski, J., Thierbach, G., and Pühler, A. (1994). Small mobilizable multi-purpose cloning vectors derived from the *Escherichia coli plasmids* pK18 and pK19: selection of defined deletions in the chromosome of *Corynebacterium glutamicum*. *Gene* 145, 69–73. doi: 10.1016/0378-1119(94)90324-7.

Simon R, Priefer U, P. A. (1983). “Vector plasmids for in-vivo and in-vitro manipulations of Gram-negative bacteria,” in *Molecular Genetics of the Bacteria-Plant Interaction*, 393pp.

Tamura, K., Stecher, G., and Kumar, S. (2021). MEGA11: molecular evolutionary genetics analysis version 11. *Mol. Biol. Evol.* 38, 3022–3027. doi: 10.1093/molbev/msab120.

Tett, A. J., Rudder, S. J., Bourdès, A., Karunakaran, R., and Poole, P. S. (2012). Regulatable vectors for environmental gene expression in *Alphaproteobacteria*. *Appl. Environ. Microbiol.* 78, 7137–7140. doi: 10.1128/AEM.01188-12.
